# Supplementary material for: Longitudinal molecular profiling elucidates immunometabolism dynamics in breast cancer
Source: Nat Commun. 2024 May 7;15:3837. doi: 10.1038/s41467-024-47932-y (PMC11076527; doi:10.1038/s41467-024-47932-y)
Supplement: Supplementary file 1 — Supplementary Information [file 41467_2024_47932_MOESM1_ESM.pdf]

SUPPLEMENTARY INFORMATION

Longitudinal molecular profiling elucidates immunometabolism dynamics in breast cancer

Kang Wang[1], Ioannis Zerdes[1,2], Henrik J. Johansson[3], Dhifaf Sarhan[4], Yizhe Sun[4], Dimitris C. Kanellis[5], Emmanouil G. Sifakis[1], Artur Mezheyeuski[6,7], Xingrong Liu[1], Niklas Loman[8,9], Ingrid Hedenfalk[9], Jonas Bergh[1,10], Jiri Bartek[5,11], Thomas Hatschek[1,10], Janne Lehtiö[3,12], Alexios Matikas[1,10], Theodoros Foukakis[1,10]

1.Department of Oncology-Pathology, Karolinska Institutet, Stockholm, Sweden.
2.Theme Cancer, Karolinska University Hospital and Karolinska Comprehensive Cancer Center, Stockholm, Sweden.
3.Department of Oncology-Pathology, Karolinska Institutet, and Science for Life Laboratory, Stockholm, Sweden.
4.Department of Laboratory Medicine, Division of Pathology, Karolinska Institutet, Stockholm, Sweden.
5.Department of Medical Biochemistry and Biophysics, Karolinska Institutet, Stockholm, Sweden.
6.Department of Immunology, Genetics and Pathology, Uppsala University, Rudbeck Laboratory, Uppsala, Sweden.
7.Molecular Oncology Group, Vall d'Hebron Institute of Oncology (VHIO), Barcelona, Spain.
8.Department of Hematology, Oncology and Radiation Physics, Lund University Hospital, Lund, Sweden.
9.Division of Oncology, Department of Clinical Sciences, Lund University, Lund, Sweden.
10.Breast Center, Theme Cancer, Karolinska University Hospital and Karolinska Comprehensive Cancer Center, Stockholm, Sweden.
11.Danish Cancer Institute, DK-2100 Copenhagen, Denmark.
12.Division of Pathology, Karolinska University Hospital and Karolinska Comprehensive Cancer Center, Stockholm, Sweden.

Supplementary Figure

Supplementary Fig.1

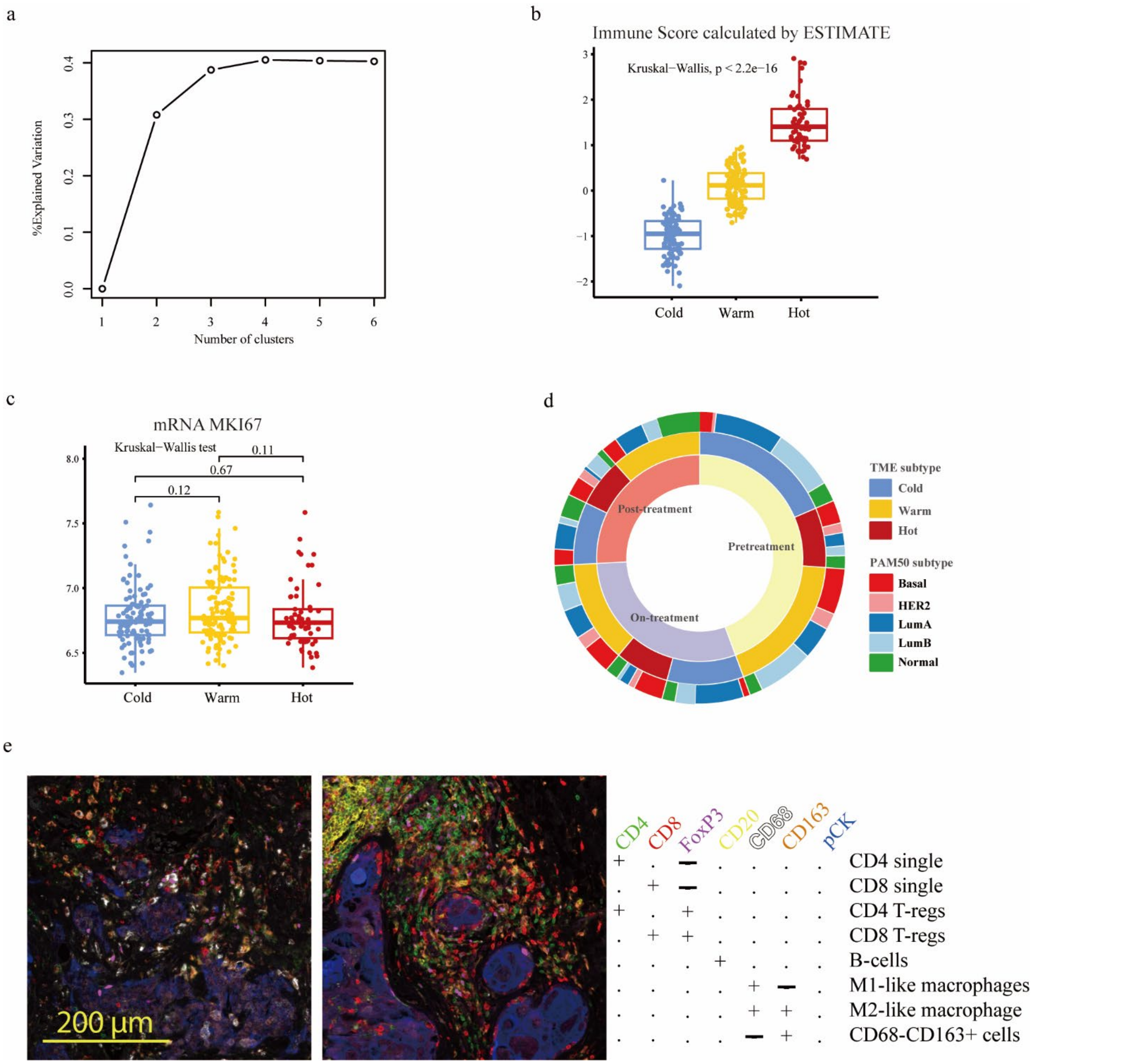

Supplementary Fig. 1 Unsupervised integrated immune classifications and immune cell definition based on mfIHC. a The association between model explained variation and the number of immune clusters, whose optimal number of clusters was determined based on the Bayesian information criterion (n.lambda=233, cpus=4). **b** Differences in ESTIMATE immune score between immune states in the PROMIX cohort. **c** Differences in mRNA MKI67 between immune states in the PROMIX cohort. **d** Overlay of immune subtypes (medium ring) with sampling timepoint and PAM50. **e** Immune cells were defined by mfIHC panel (stained for lymphocytic, macrophage and epithelial markers, i.e CD4, CD8, CD20, CD163, CD68, FoxP3 and Cytokeratin), where spectral unmixing and cut-offs for marker positivity were applied as previously described[1].

Supplementary Fig.2

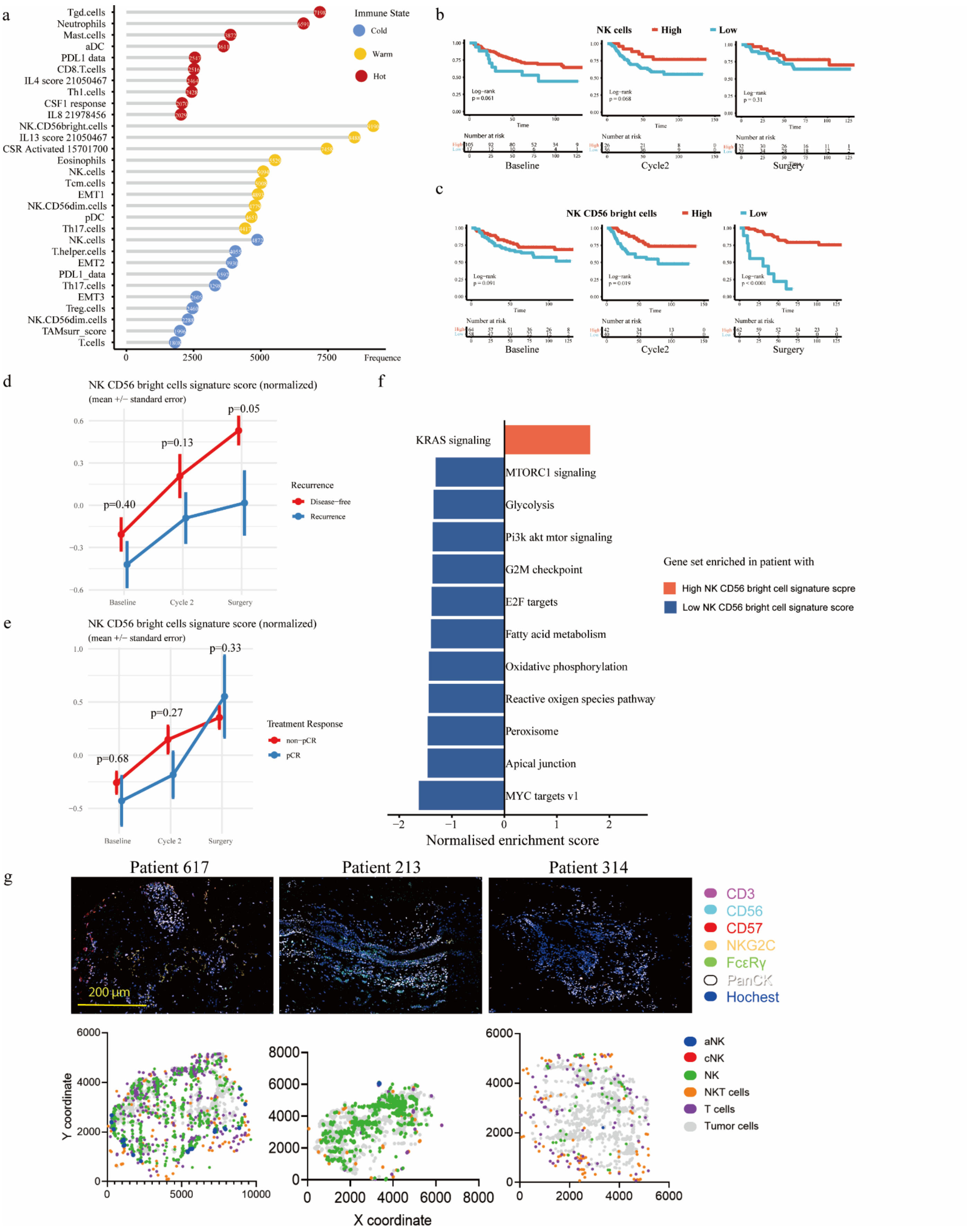

Supplementary Fig. 2 Prognostic immune biomarkers and NK cell mIF panel. a Frequency of immune gene signatures selected by Lasso-bootstrapping highlighted the NK cell contributing to predictive DFS model within each immune state subgroup. **b,c** Disease-free survival analysis of

patients according to tumors' NK cell and NK CD56^{bright} cells at three sampling timepoints, respectively. **d,e**, NK CD56^{bright} cell gene signature score comparisons between patients with and without disease recurrence/pCR by timepoints. P values were derived from wilcoxon rank sum test. **f**. Gene set enrichment analysis (GSEA) based on differential genes between high and low NK CD56^{bright} cell gene signature scores. **g** mIF NK cell panel (stained for lymphocytic, NK cells and epithelial markers, i.e CD56, CD57, NKG2C, FcεR, CD3 and Hochest) showed that NK cells are mostly located in patients' surgical specimens, who had long-term disease-free survival. According immune cells were defined as following: aNK cells: CD3-CD56+CD57+NKG2C+FcεRγ-; cNK cells: CD3-CD56+CD57+FcεRγ+; NK cells: CD3-CD56+; T cells: CD3+ CD56-; NKT cells: CD3+CD56+; Tumor cells: PanCK+. DFS, disease-free survival; mIF, multiplex immunofluorescence; aNK, adaptive NK cells; cNK, conventional NK cells.

Supplementary Fig. 3

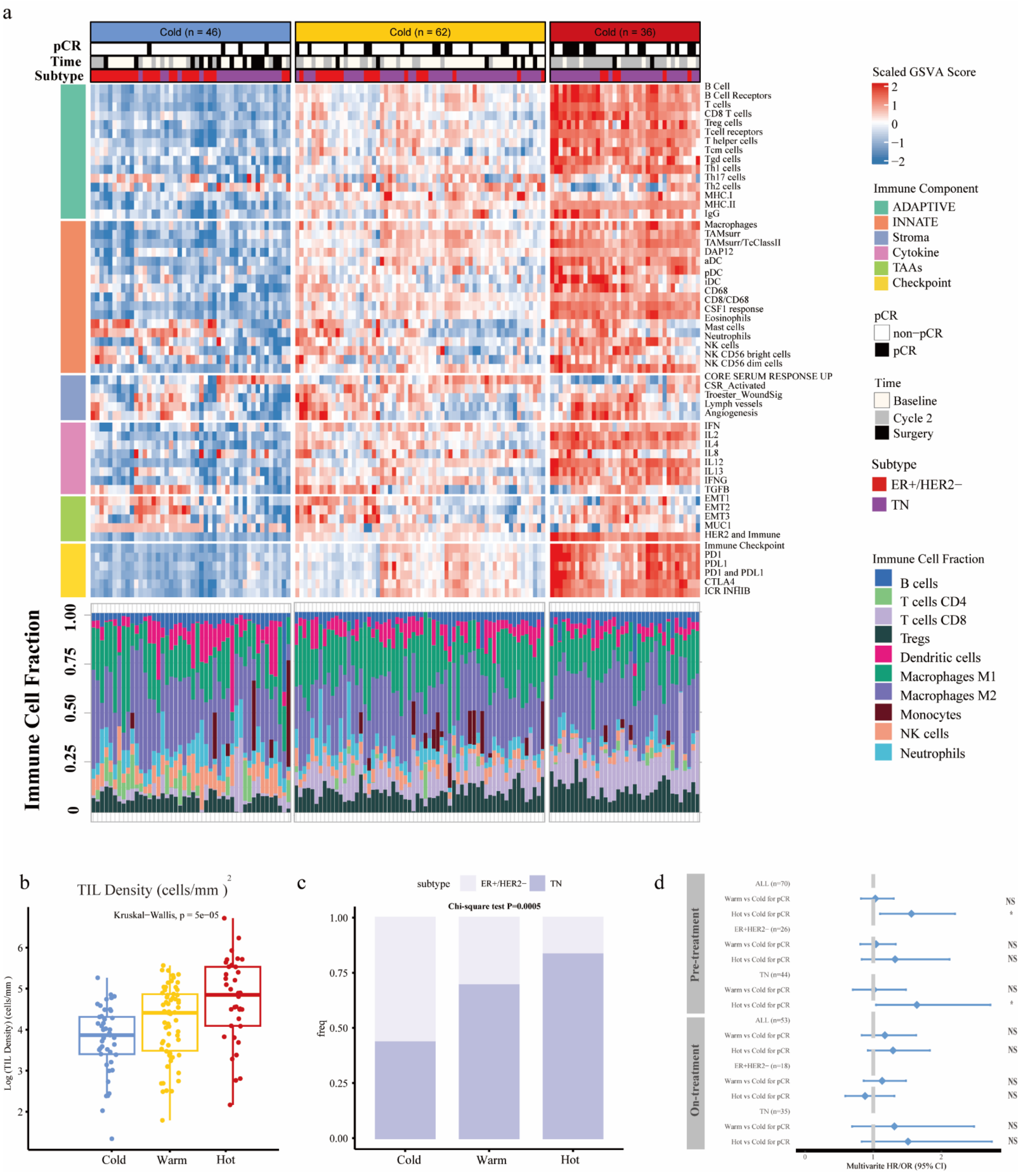

Supplementary Fig. 3 External validation of immune classifications associated with clinical outcomes. a Unsupervised clustering using a joint latent variable model reveals distinct subtypes of breast cancer based on immune gene signatures and immune cell composition in Korean NAC cohort[2]. **b** TILs density between three immune states. **c** Distribution of breast cancer subtype among the immune states. **d** Forest plot of multivariate Logistic regression analysis for pCR by sampling timepoint and breast cancer subtype, adjusting for tumor size and lymph node status. The odds

ratios are shown with 95% confidence intervals. **p < 0.05; *p < 0.1; NS p > 0.05. TILs, tumor-infiltrating lymphocytes; pCR, pathologic complete response.

Supplementary Fig. 4

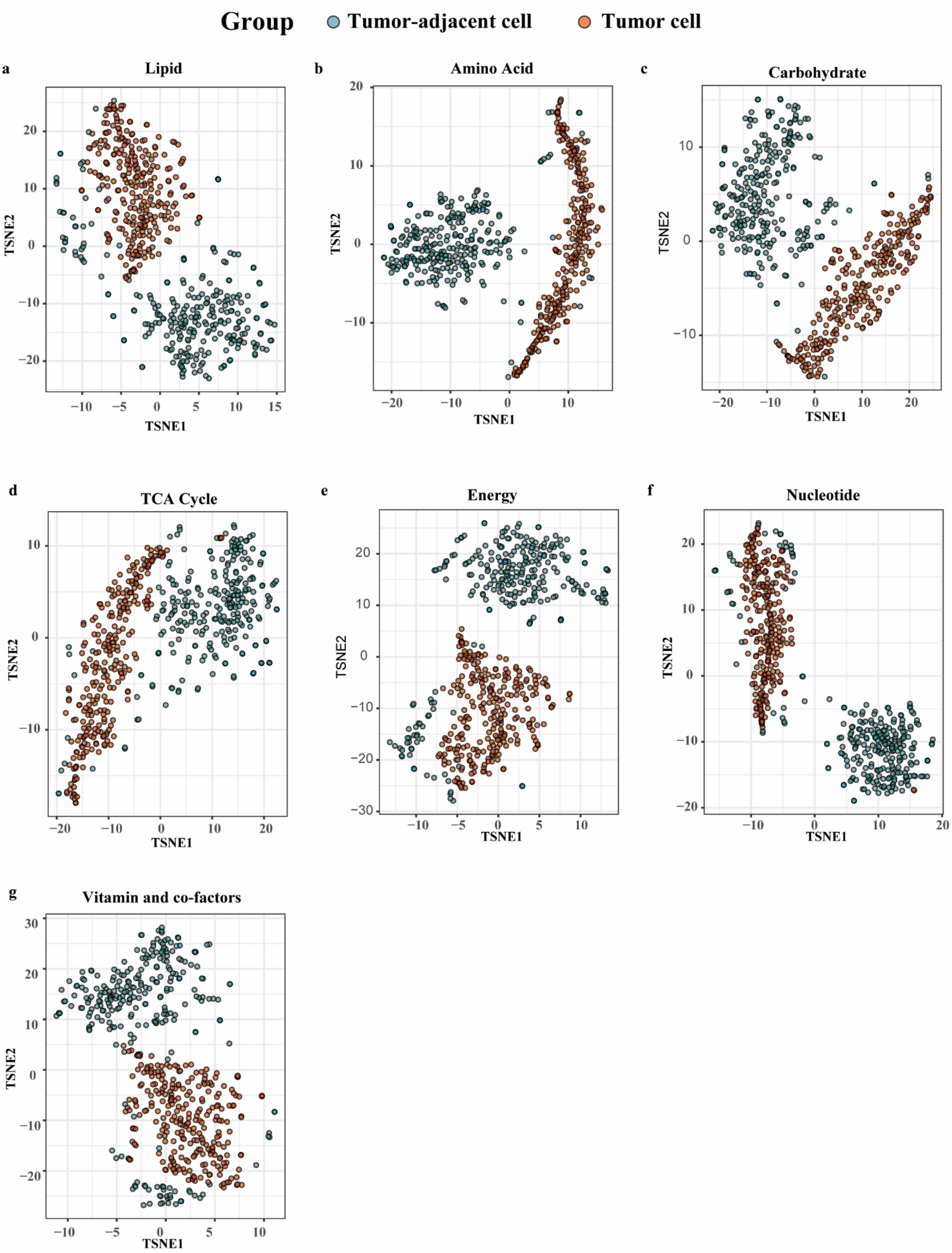

Supplementary Fig. 4 t-SNE plot visualizing metabolic cluster assignments of tumor-adjacent and tumor cells-based gene expression profiling. T-distributed stochastic neighbor embedding

(tSNE) analysis of PROMIX samples according to tumor-adjacent and tumor cells-based gene expression profiling involved in **a** Lipid, **b** Amino acid, **c** Carbohydrate, **d** TCA Cycle, **e** Energy, **f** Nucleotide, and **g** Vitamin and co-factors.

Supplementary Fig. 5

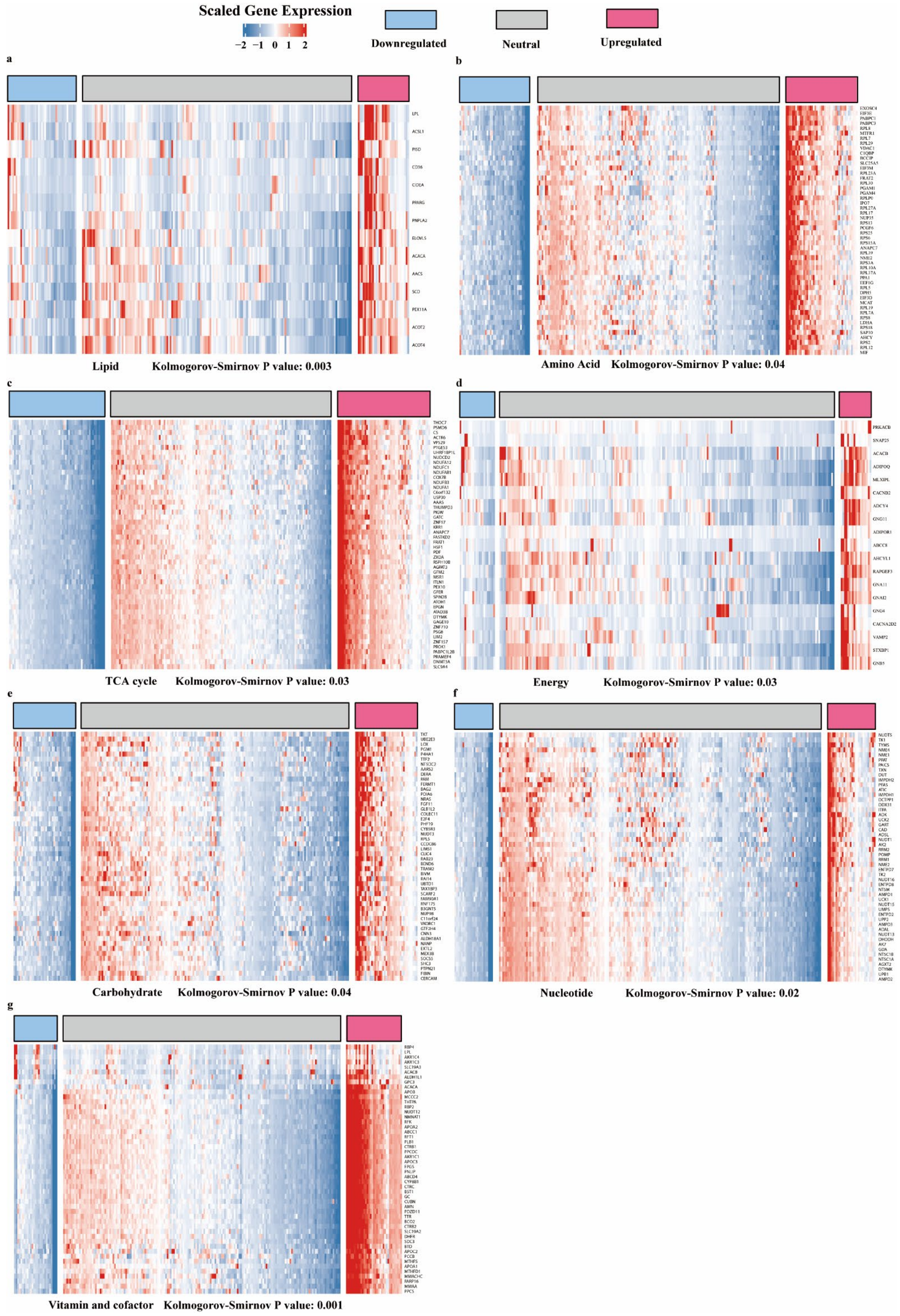

Supplementary Fig. 5 Heatmap of differential tumor-cell based metabolic genes between metabolic phenotype. Significant tumor-cell based DGE (FDR<0.1) are shown in **a** Lipid, **b** Amino acid, **c** Carbohydrate, **d** TCA Cycle, **e** Energy, **f** Nucleotide, and **g** Vitamin and co-factors, where Kolmogorov-Smirnov test indicated that FDR values of each metabolic pathway are lower than those from other coding genes (all P<0.05).

Supplementary Fig. 6

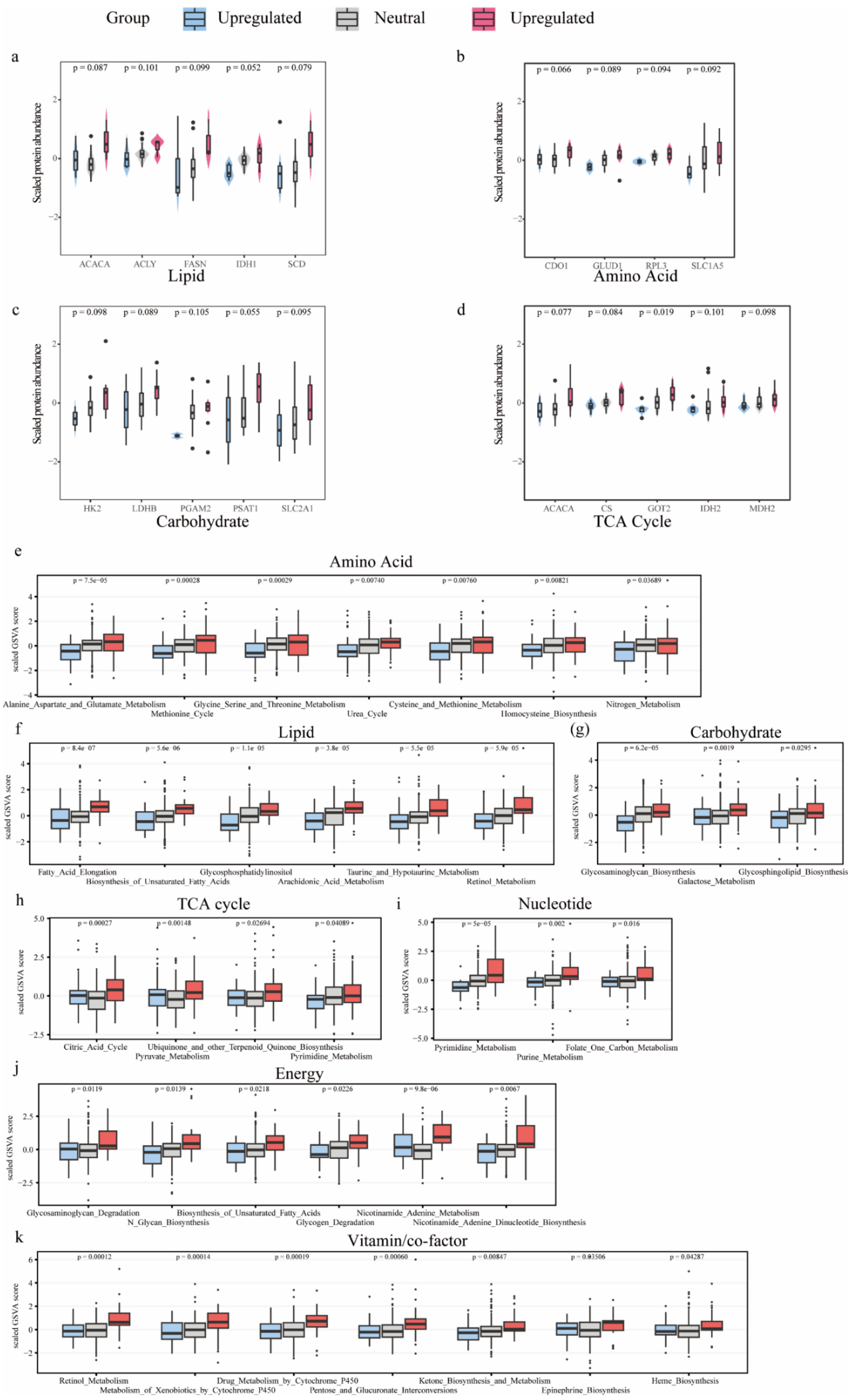

Supplementary Fig. 6 Metabolic protein abundance between metabolic phenotype. Comparison of representative metabolic proteins among three metabolic phenotypes in **a** lipid, **b**

amino acid, **c** carbohydrate, and **d** TCA Cycle. Comparison of representative KEGG metabolic phenotype among three metabolic phenotypes in **e** amino acid, **f** lipid, **g** carbohydrate, **h** TCA cycle, **i** nucleotide, **j** energy, **k** vitamin/co-factor. P values were derived from Kruskal–Wallis tests.

Supplementary Fig. 7

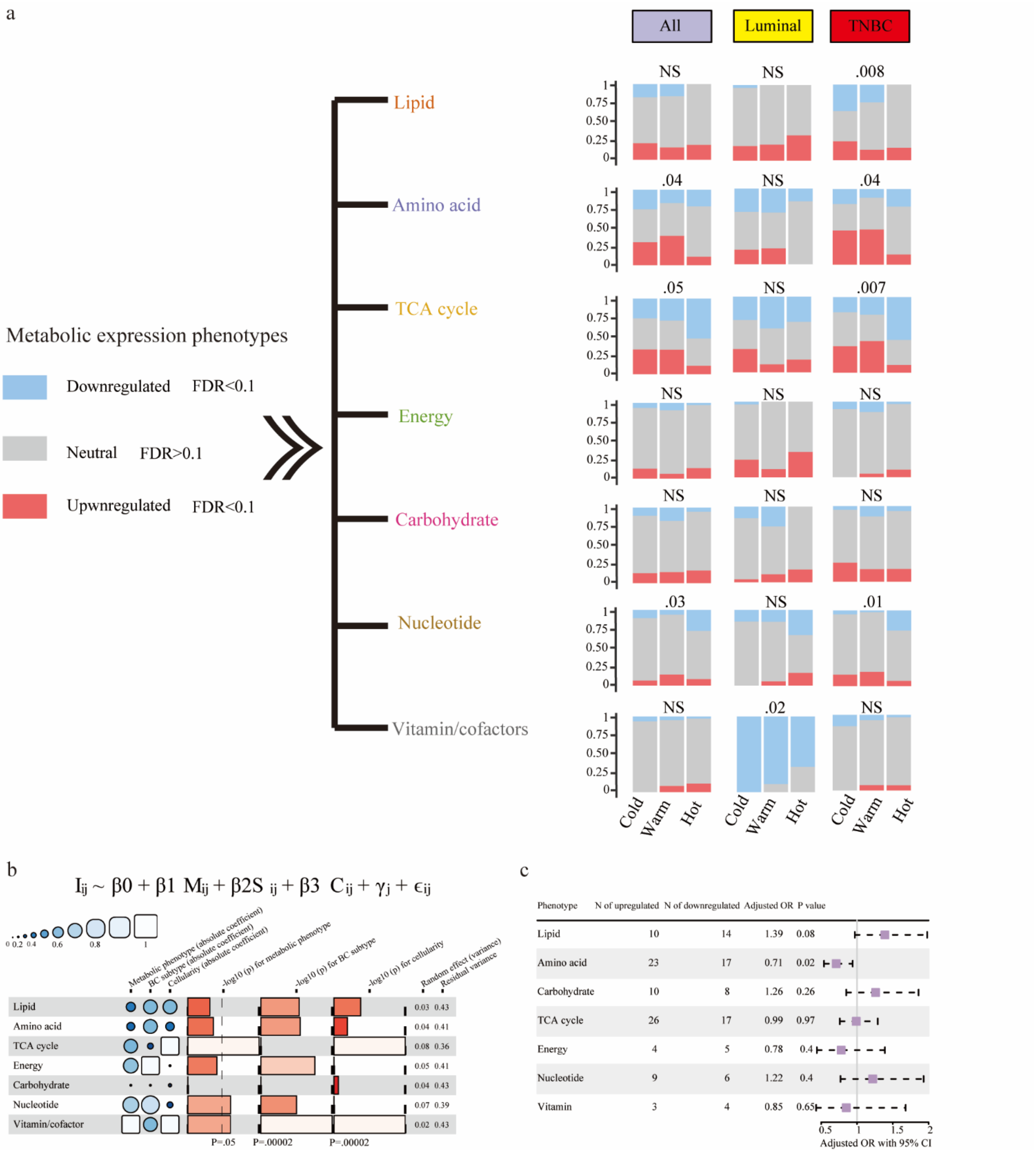

Supplementary Fig. 7 Bulk gene expression profiling based metabolic phenotypes interact with immune subtypes in Korean NAC cohort[2]. **a** Percentage stacked bar chart showed distribution of bulk gene expression based metabolic phenotype in seven pathways on different immune state, where P values were derived from Chi-Square test or Fisher's exact test. b Funkyheatmap depicting the coefficient, random term, residual variance and p value of LEME, which was conducted within all samples to identify interaction effects between immune state (I) and bulk tumor metabolic phenotypes (M), adjusting for the breast cancer subtype (S) and cellularity (C). c Forest plot depicting association between bulk metabolic phenotype and pCR. LMEM, linear mixed-effects model; pCR, pathologic complete response.

Supplementary Fig. 8

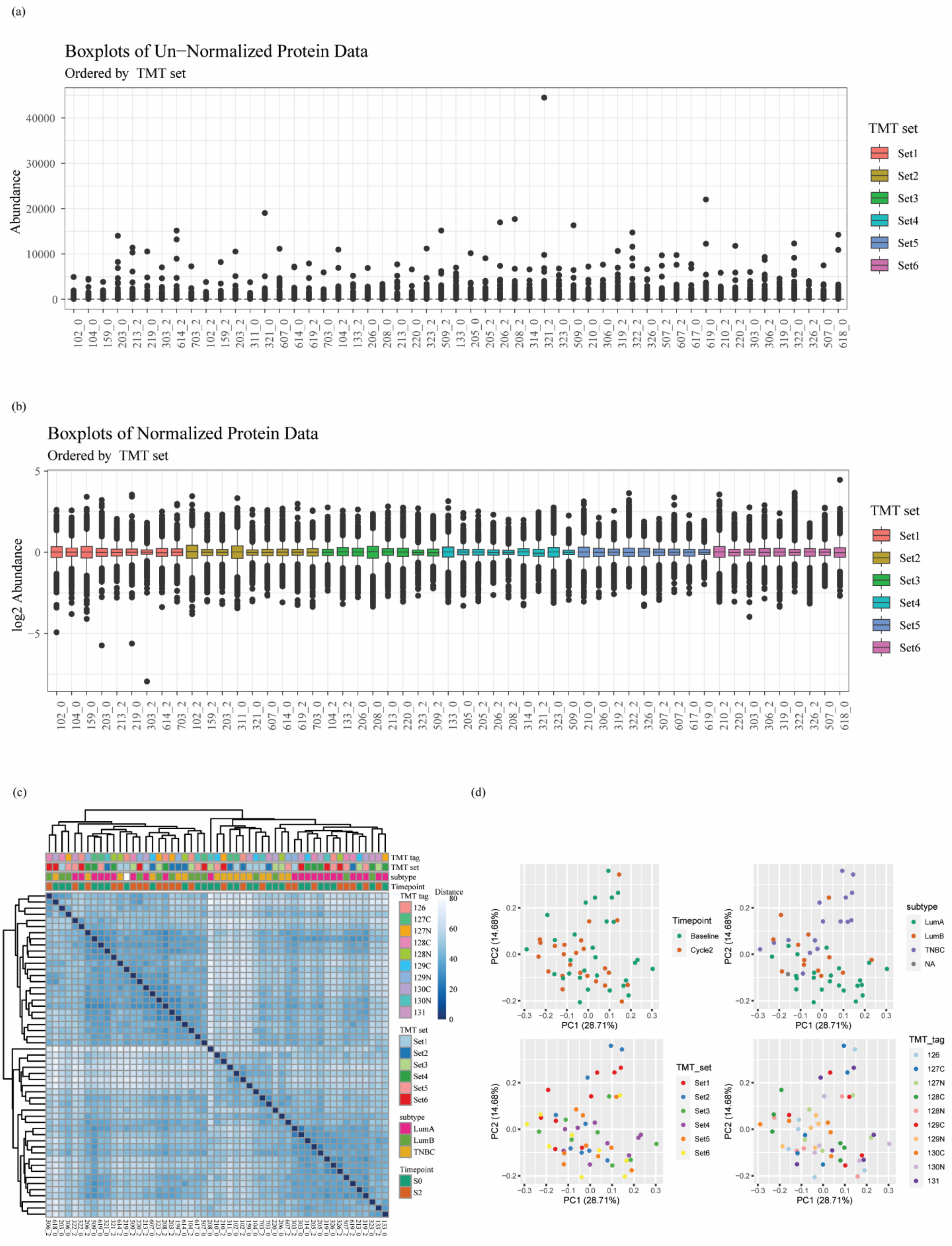

Supplementary Fig. 8 Quality control of mass spectrometry (MS) data. Boxplot show unnormalized (**a**) and normalized (**b**) protein abundance by TMT set. **c**. Heatmap depicting euclidean distance of samples calculated by mass spectrometry data. **d**. Principal component analysis by treatment timepoint, subtype, TMT set and TME tage, respectively.

Supplementary Fig. 9

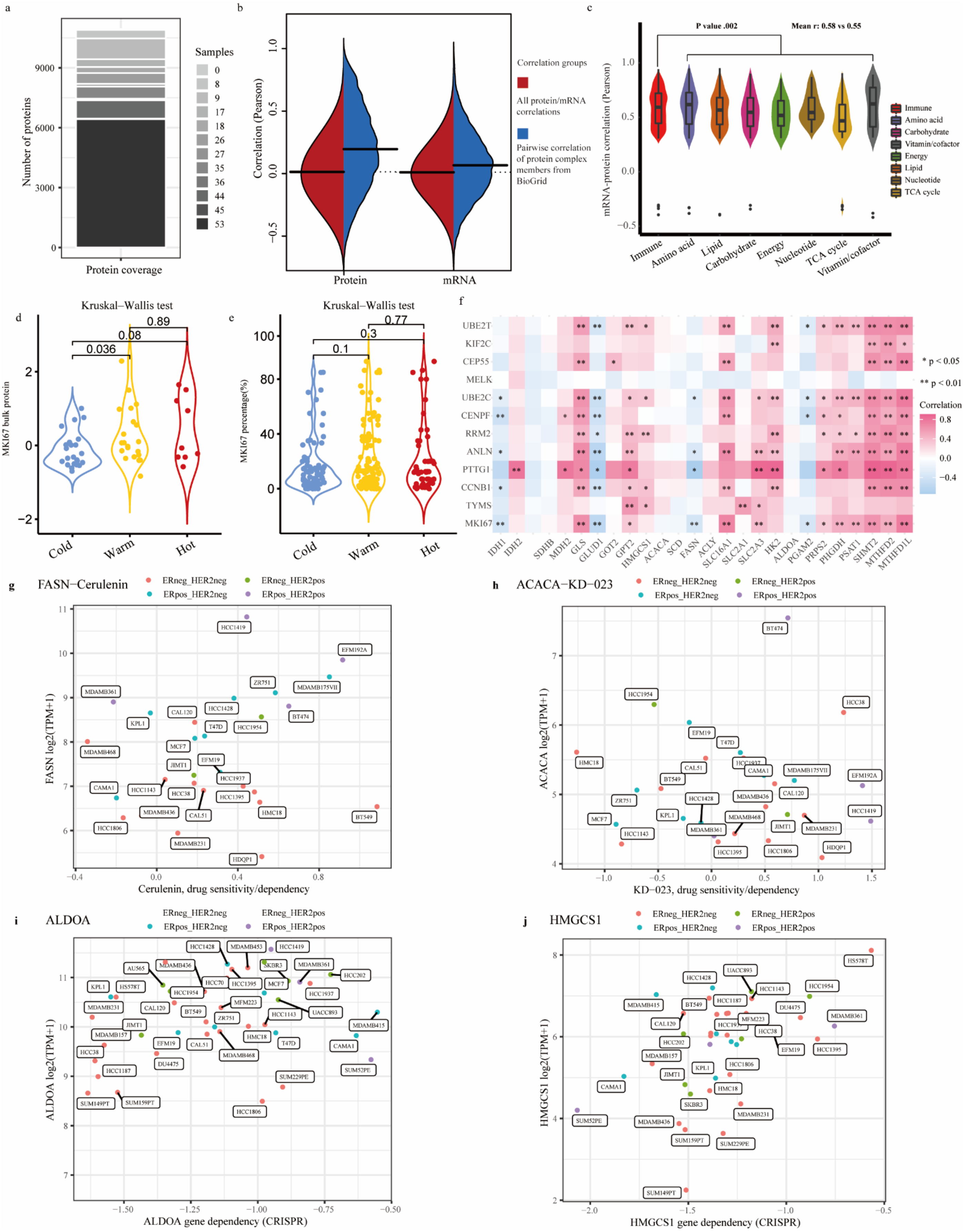

Supplementary Fig. 9 Metabolic protein abundance across the immune state. a Protein coverage of MS-based proteomics. **b** Comparison of all pairwise correlations to correlations from known interaction pairs from Biogrid database, using quantitative protein and RNA levels across the 53 tumors. **c** Comparison of all RNA-protein pairwise correlations across immunometabolic

categories. **d** Comparison of MKI67 protein from MS-based proteomic across TME subtype. **e** Comparison of IHC MKI67 percentage score that is defined as the percentage of positively stained tumor cells among the total number of malignant cells assessed, across TME subtype. **f** Correlation matrix of proliferation proteins (row) and metabolic proteins (column), where p values and Pearson's r were indicated in heatmap. **g,h** Scatter plot showing breast cancer cell lines that have metabolic vulnerability (x-axis) and respond to its inhibitor (y-axis) based on DepMap portal. **I,j** Scatter plot showing breast cancer cell lines with potential metabolic targets (high gene expression and highly essential genes).

Supplementary Fig. 10

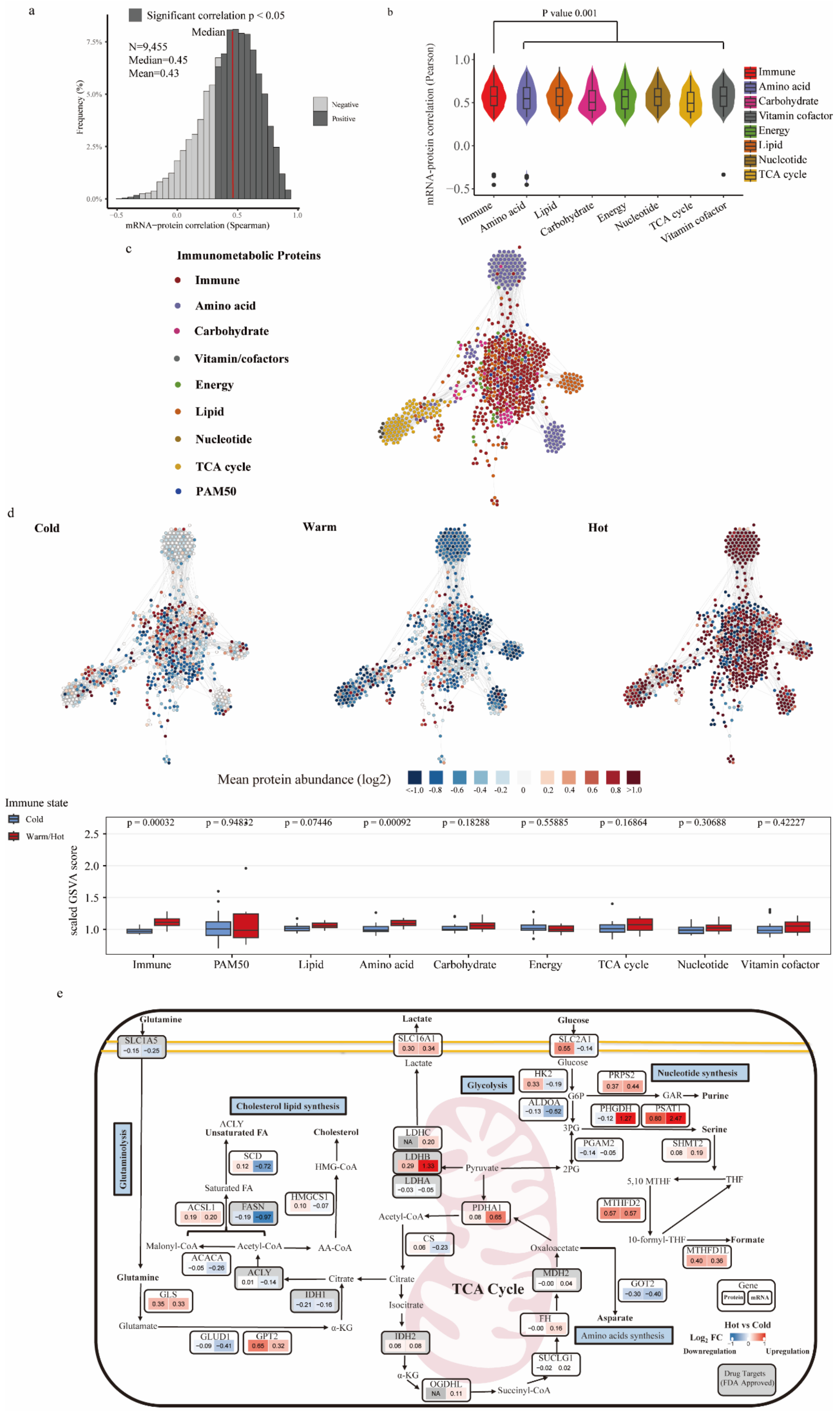

Supplementary Fig. 10 MS-based proteomic landscape of immunometabolic phenotype and pathways in Oslo2 cohort[3]. **a** Correlation between protein and mRNA quantitative values (Spearman) of individual genes. **b** Comparison of all RNA-protein pairwise correlations across immunometabolic categories. **c** Breast cancer protein correlation network based on immunometabolic and PAM50 proteins (n=2,837 in total) using > 0.3 Pearson correlation and KCore > 2 cutoff. **d** Visualization of average proteome quantification of breast cancer immune subtype in the correlation network. Boxplot showing difference of mean protein abundance of each module between cold and warm/hot tumors. Statistical significance (P value) was determined using Wilcoxon signed-rank test. **e** Pathway diagram summarizing metabolic genes involved in the TCA cycle, glycolysis, nucleotide, amino acid, and cholesterol lipid synthesis and metabolism. Alterations are defined by significant upregulation or downregulation of protein abundance (left) and mRNA expression (right) between hot and cold tumors (expressed as log2(fold-change)). Red, upregulated genes/proteins in immunological hot tumor; blue, downregulated genes in immunological hot tumor. The grey panel highlighted the FDA approved drug targets.

Supplementary Fig. 11

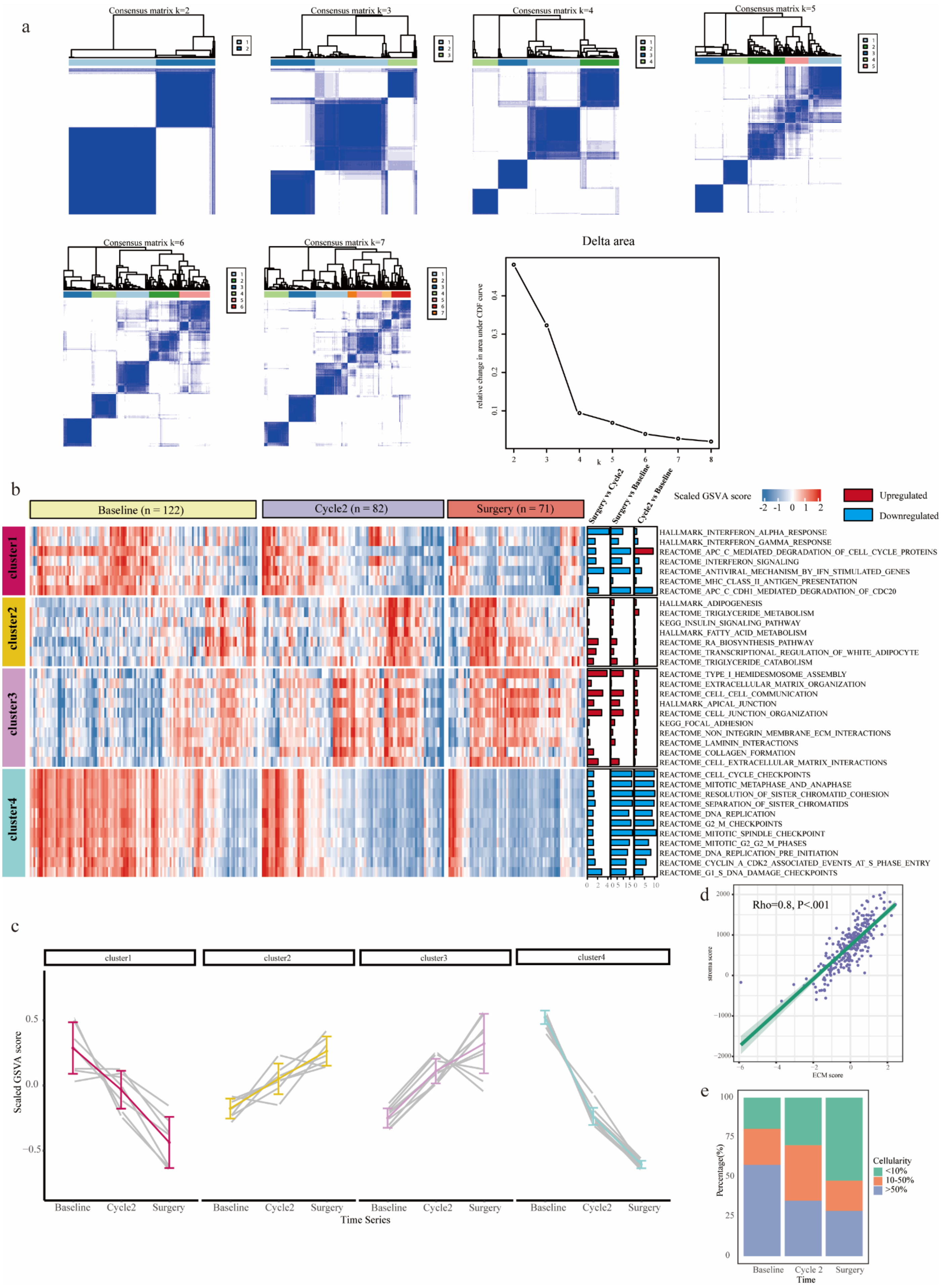

Supplementary Fig. 11 Transcriptome response to neoadjuvant chemotherapy. a Consensus matrix and cumulative distribution function (CDF) plot identified the optimal number (n=4) of the consensus cluster based on pair-wise differential expressed genes between sampling timepoint. **b** Expression patterns of representative pathways (a) mapped to the four differential expressed gene clusters, which involved immune response (C1), metabolism (C2), extracellular matrix (ECM) (C3), and tumor proliferation (C4) pathways. GSVA scores in **b** were centered and scaled to z-scores for each pathway geneset. Statistical significance of differential expressed was determined using linear mixed-effects regression analysis (LMER) and shown as −log10(p-value) based on the sign of the t-statistics. **c** Aggregate expression patterns of pathways mapped to the three differential expressed clusters in the PROMIX trial. Gray lines represent individual gene signatures (GSVA scores) and vertical lines represent the averages for each sample group. Error bars represent the standard deviation of the scaled GSVA score at each time point. **d** Correlation between stroma score derived from ESTIMATE and extracellular matrix (ECM) GSVA score. E Distribution of cellularity categories across time point.

Supplementary Fig. 12

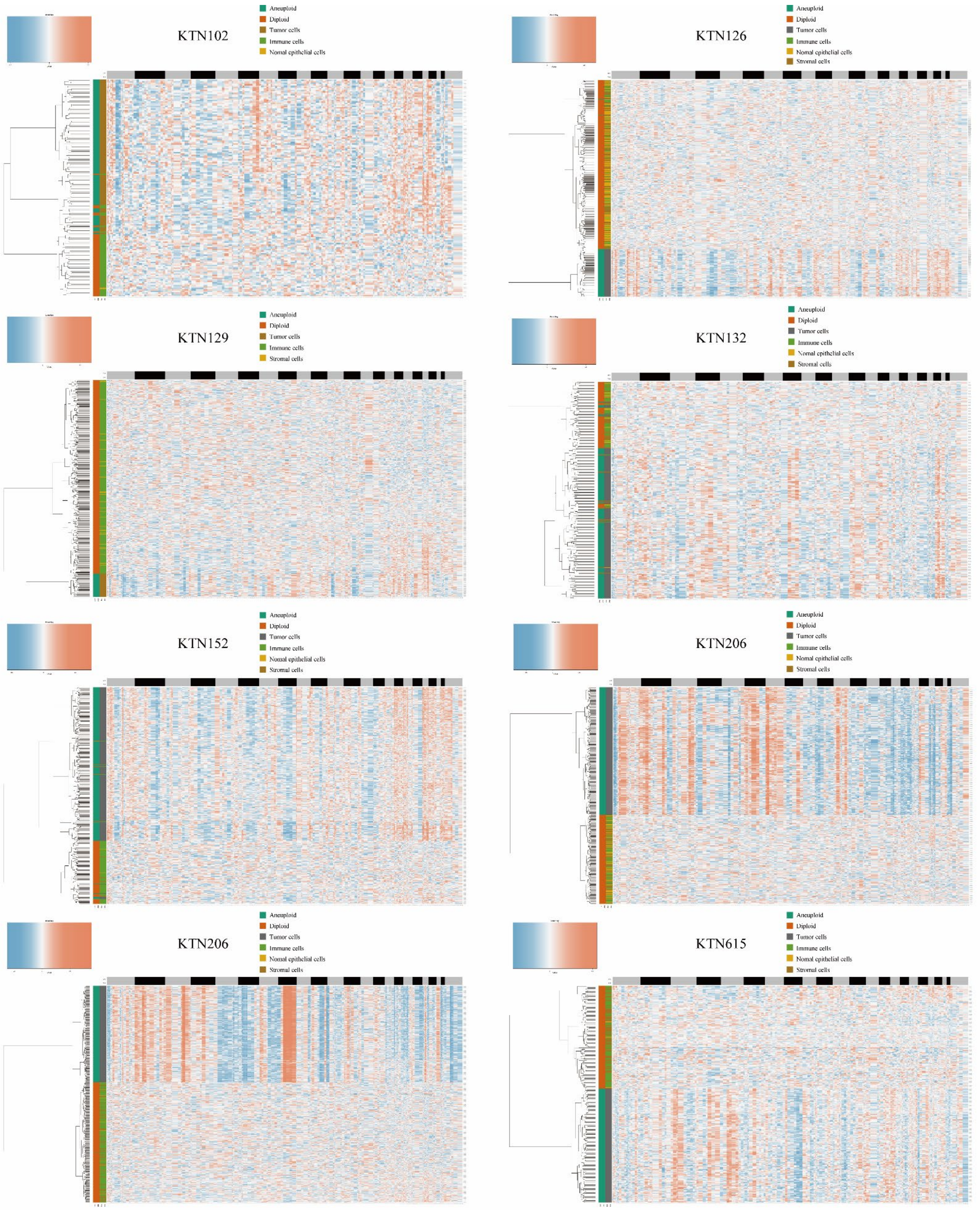

Supplementary Fig. 12 Clustered heat maps of single-cell copy number profiles estimated by CopyKAT from snRNA-seq data for longitudinal breast cancer samples in PROMIX trial.

Supplementary Fig. 13

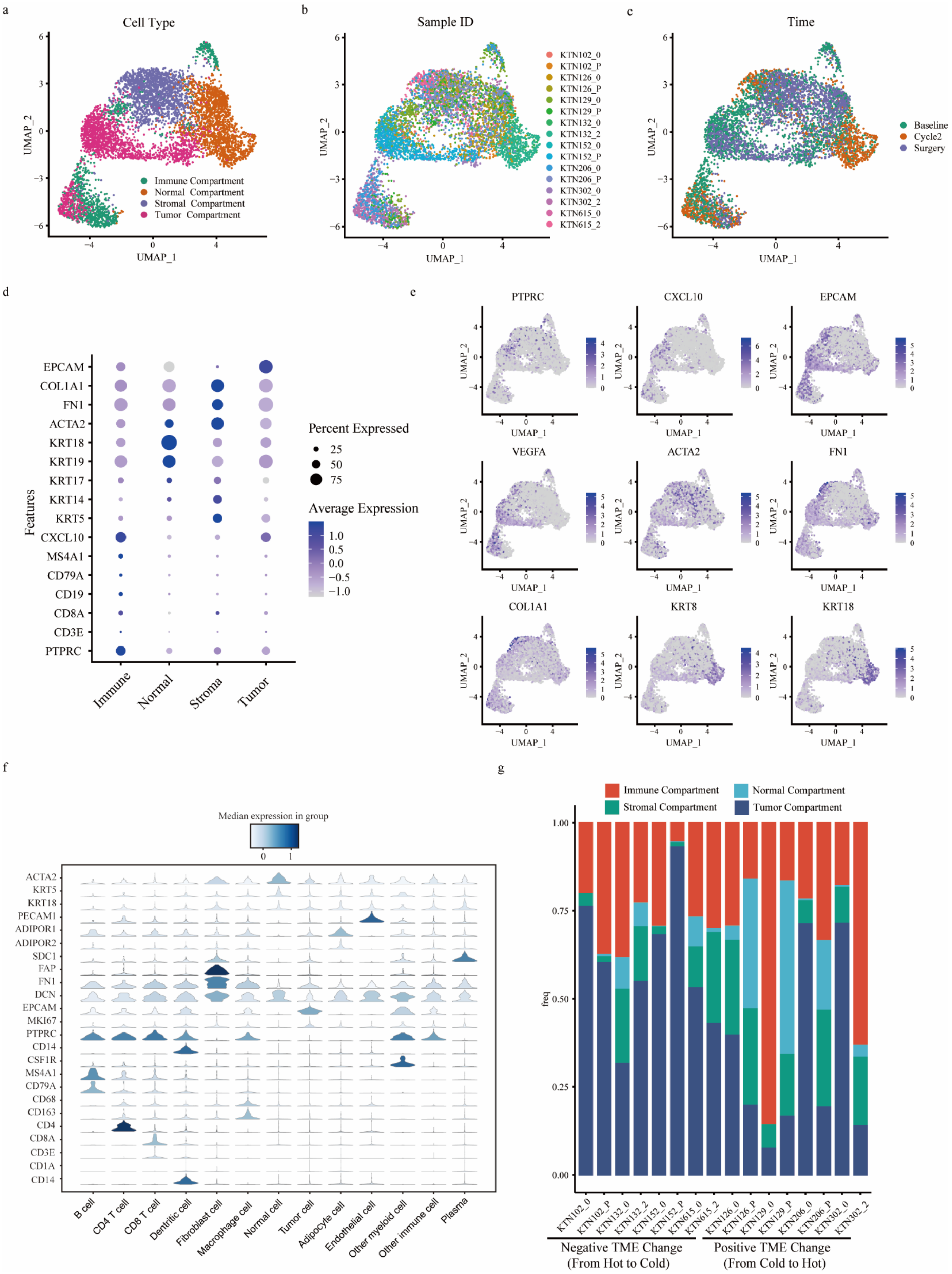

Supplementary Fig. 13 snRNA-seq defines the cellular ecosystem of breast cancer samples from PROMIX trial. **a,b,c** Uniform manifold approximation and projection (UMAP) of snRNA-seq of all 16 samples colored by compartment (a), sample (b), and sampling time point (c). **d** Bubble plot shows the compartment-type marker gene expression levels. **e** UMAPs show the compartment-type marker gene expression levels. **f** Stacked violin plot shows the cell-type marker gene expression levels. **g** Distribution of cellular compartment between patients within TME change group. TME, tumor microenvironment.

Supplementary Fig. 14

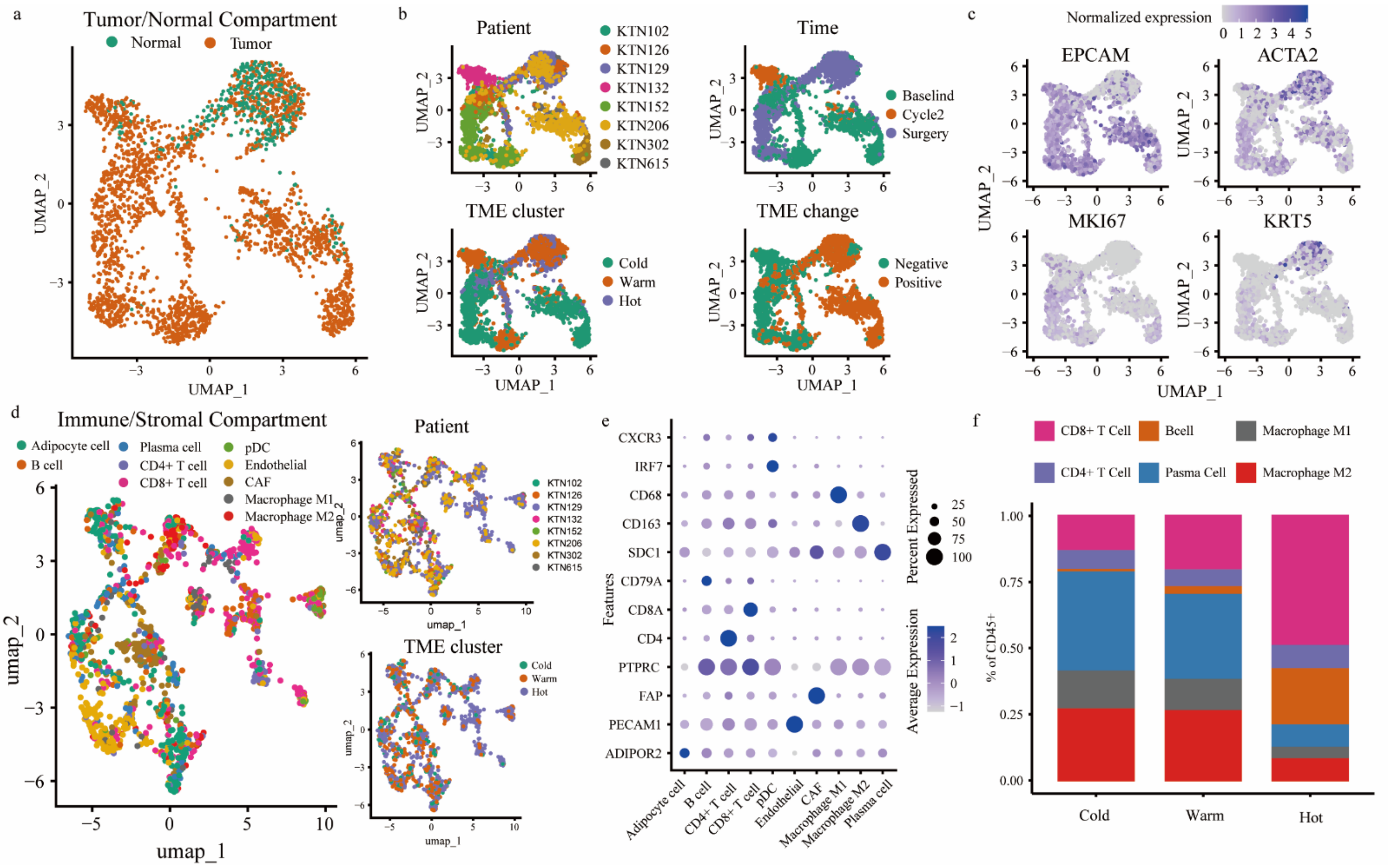

Supplementary Fig. 14 Breast epithelial, immune cell, and stromal cells from breast cancer samples in PROMIX trial. **a,b** Uniform manifold approximation and projection (UMAP) of snRNA-seq of breast epithelial cells colored by compartment (a), patient, sampling time point, TME subtype, TME change (b). **c** Marker gene expression of representative breast cancer/normal epithelial cell markers. **d** UMAP of snRNA-seq of immune and stromal compartments colored by cell types. **e** Bubble plot shows the immune and stromal cell marker gene expression levels. **f** Proportion of immune cells within different TME subtype. TME, tumor microenvironment.

Supplementary Fig. 15

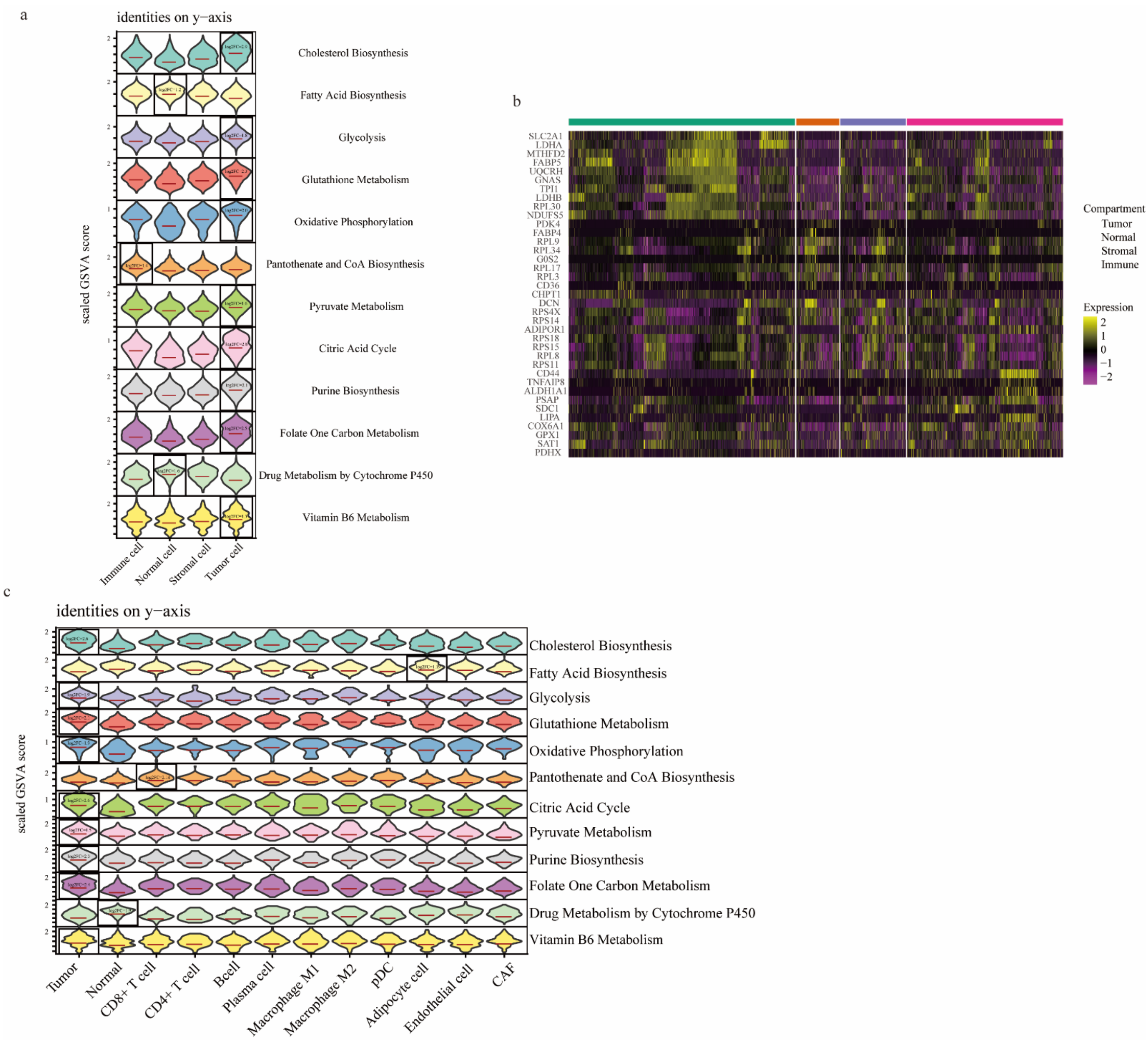

Supplementary Fig. 15 snRNA-seq reveals metabolism profiles across cellular compartment. a GSVA score of reprehensive metabolic gene signature across cellular compartments. **b** Heatmap of the expression levels of the top 10 differentially expressed genes in metabolism among tumor, normal, immune and stromal compartments. **c** Stacked violin plot shows the representative metabolic gene signature scores across different cell types.

Supplementary Fig. 16

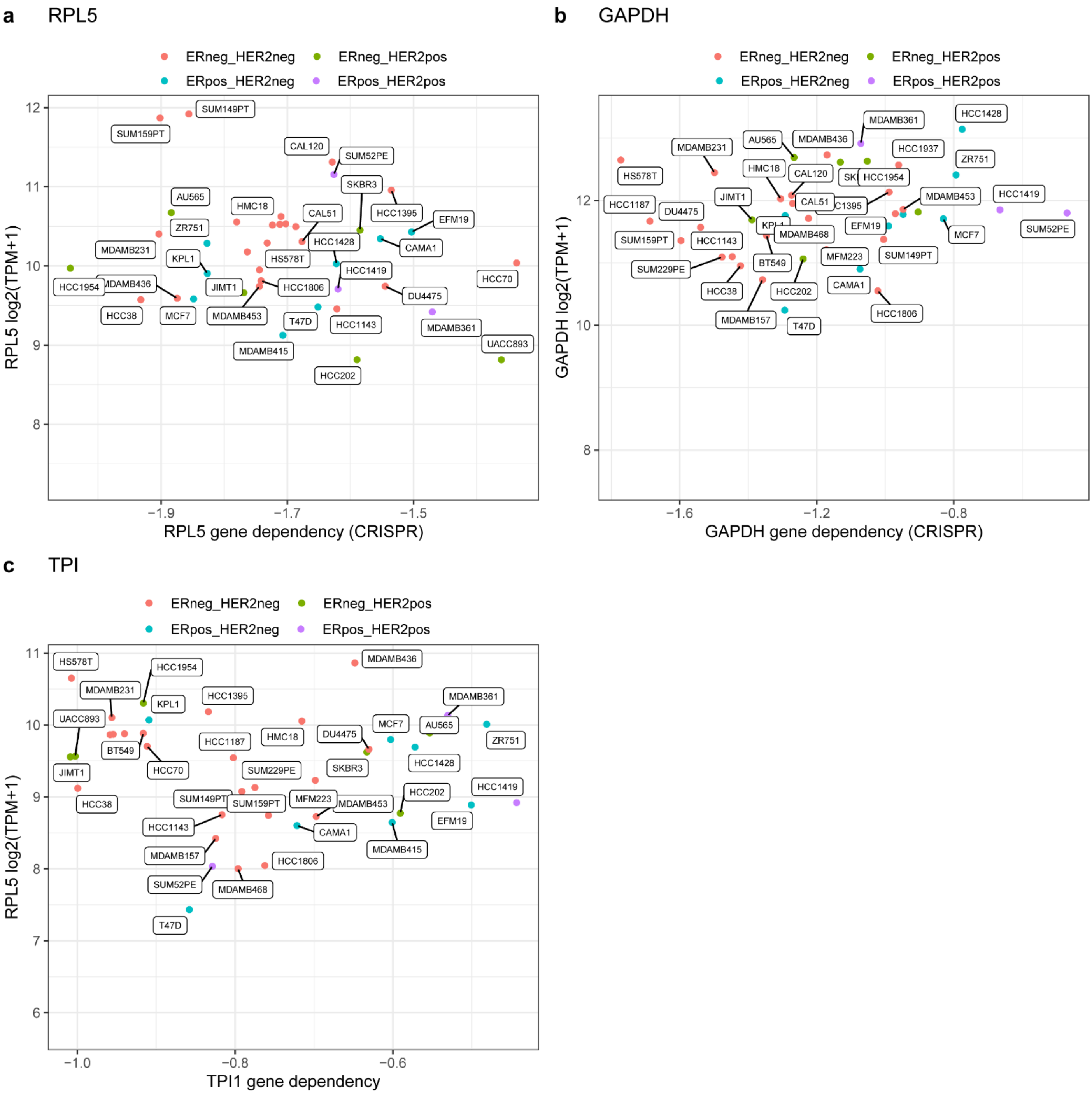

Supplementary Fig. 16 Functional analyses on metabolic drivers identified in snRNA-seq analyses. a,b,c Scatter plots showing target potential within breast cancer cell lines (high gene expression associated with high gene dependency) in DepMap 21Q2.

Supplementary Fig. 17

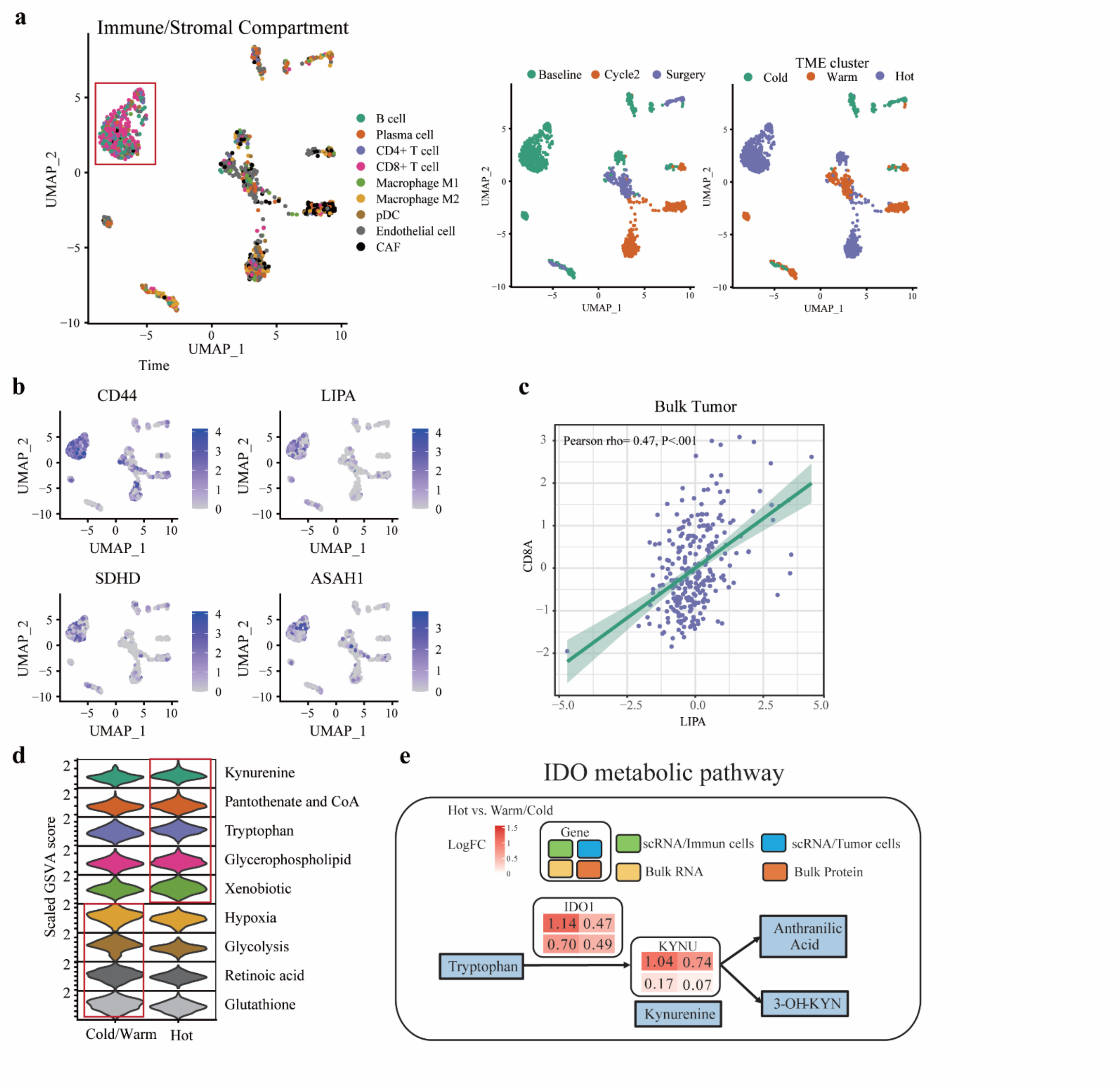

Supplementary Fig. 17 Metabolic biomarkers within immune cells. a Uniform manifold approximation and projection (UMAP) of snRNA-seq shows metabolic clusters of immune and stromal cells, which was colored by sampling time point and TME subtype. **b** Representative upregulated metabolic genes in baseline immune cells in the hot TME, which is indicated with red block in **a**. **c** Correlation between LIPA and CD8A in bulk gene expression data in PROMIX. **d** Stacked violin plot shows difference of representative metabolic signature score between immune cells within hot and cold/warm TME. **e** Pathway schema of IDO metabolism shows the differently expressed genes between hot and cold/warm tumors.

Supplementary Fig. 18

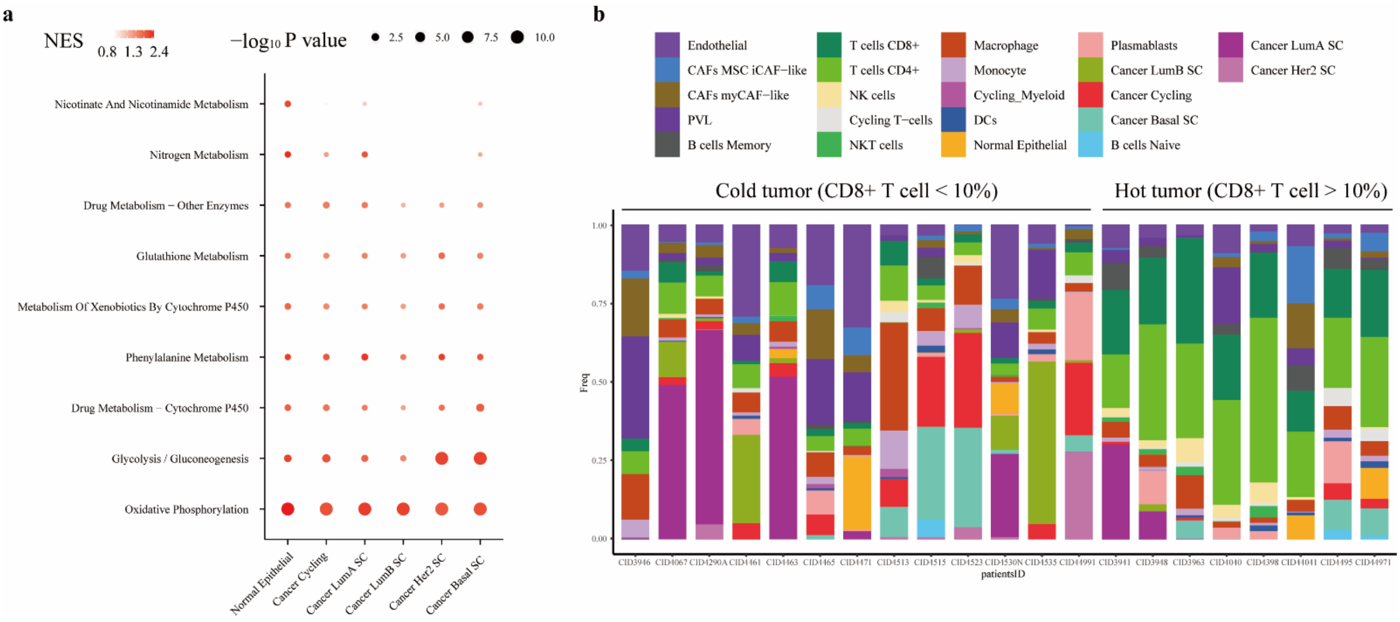

Supplementary Fig. 18 Metabolic heterogeneity in TME analyzed based on the scRNA-seq cohort[4]. Metabolic pathways enriched in genes with highest contribution to the metabolic heterogeneities among malignant cells (**a**). **b**. The cell proportion of hot and cold tumors. The single epithelial cell subpopulation was previously annotated[5] (Sunny Z. Wu et al, Supplementary Table 4. scSubtype gene lists).

Supplementary Fig. 19

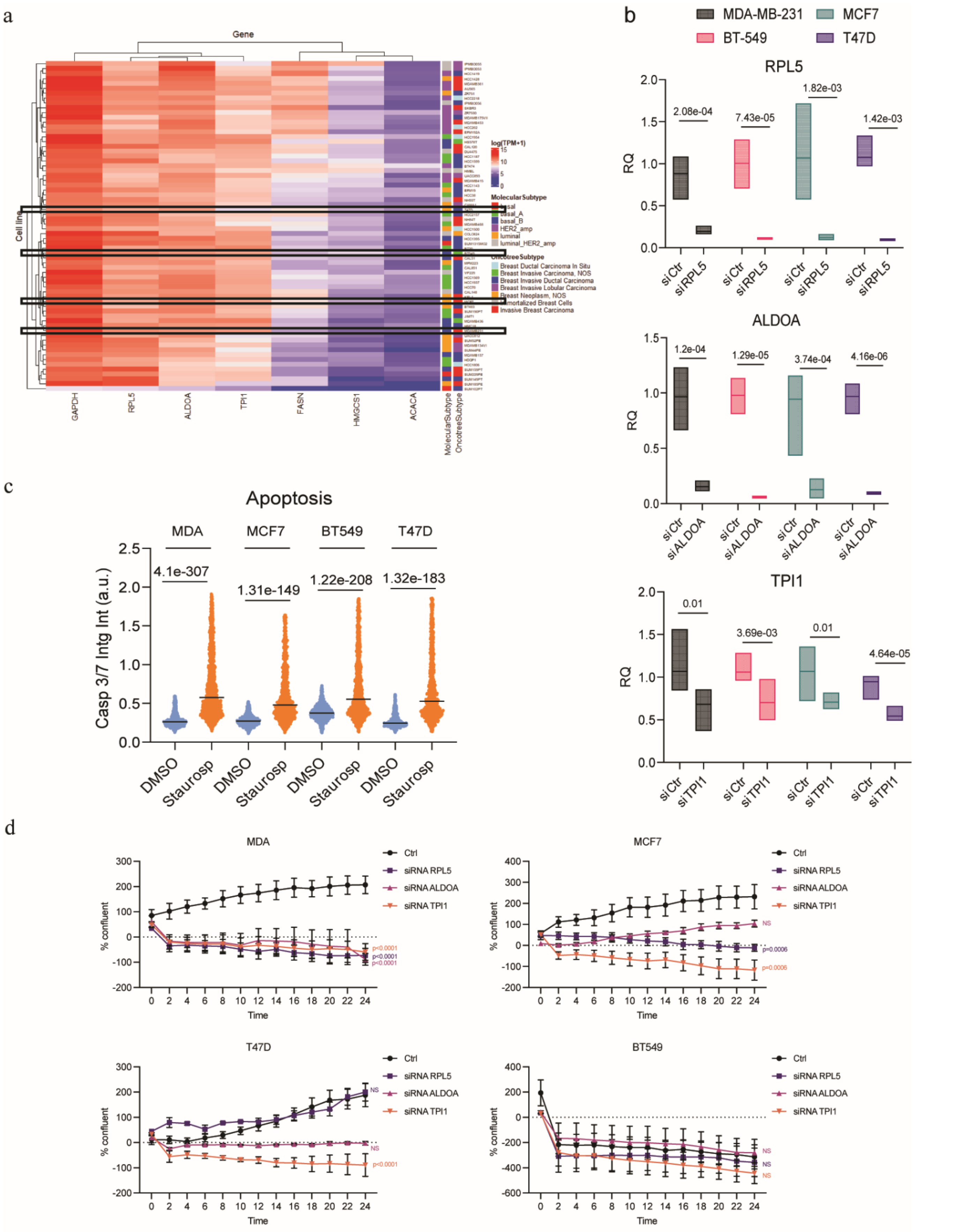

Supplementary Fig 19: **a.** Heatmap of baseline transcriptomic expression of different metabolic-related genes (columns) in various breast cancer cell lines (rows), the breast cancer cell lines selected for the *in vitro* studies are highlighted within the black-outlined boxes. **b**. Relative quantitation of RPL5, ALDOA and TPI1 mRNA levels following their siRNA-mediated depletion in

two luminal (MCF7, T47D) and two basal (MDAMB231, BT549) breast cancer cell lines. All qPCR assays were performed three independent times in triplicates (N = 9). Data are shown as mean ± SD, Students t-test (treatments compared to siCtr). **c**. Cell apoptosis is shown as arbitrary units of Caspase 3/7 integrated intensity (immunofluorescence) following treatment of various breast cancer cell lines with 2µM Staurosporine for 24 hours. The apoptotic assay was performed twice with a total of N = 1000-2000 cells counted for each experimental condition. Data are shown as mean ± SD, Students t-test (treatments compared to DMSO). **d.** Tumor cell confluence after following T-cell co-culture in control cell lines and upon knock-down of RPL5, ALDOA or TPI1 for 24hours and 2-hours time-lapse using live cell imaging. Cumulative data (n=4) are shown as mean ± SEM, experiments were repeated twice with three technical replicates and eight biological replicates. Statistical analyses were performed using Dunnett's multiple comparisons test presenting adjusted p-values.

Supplementary Fig. 20

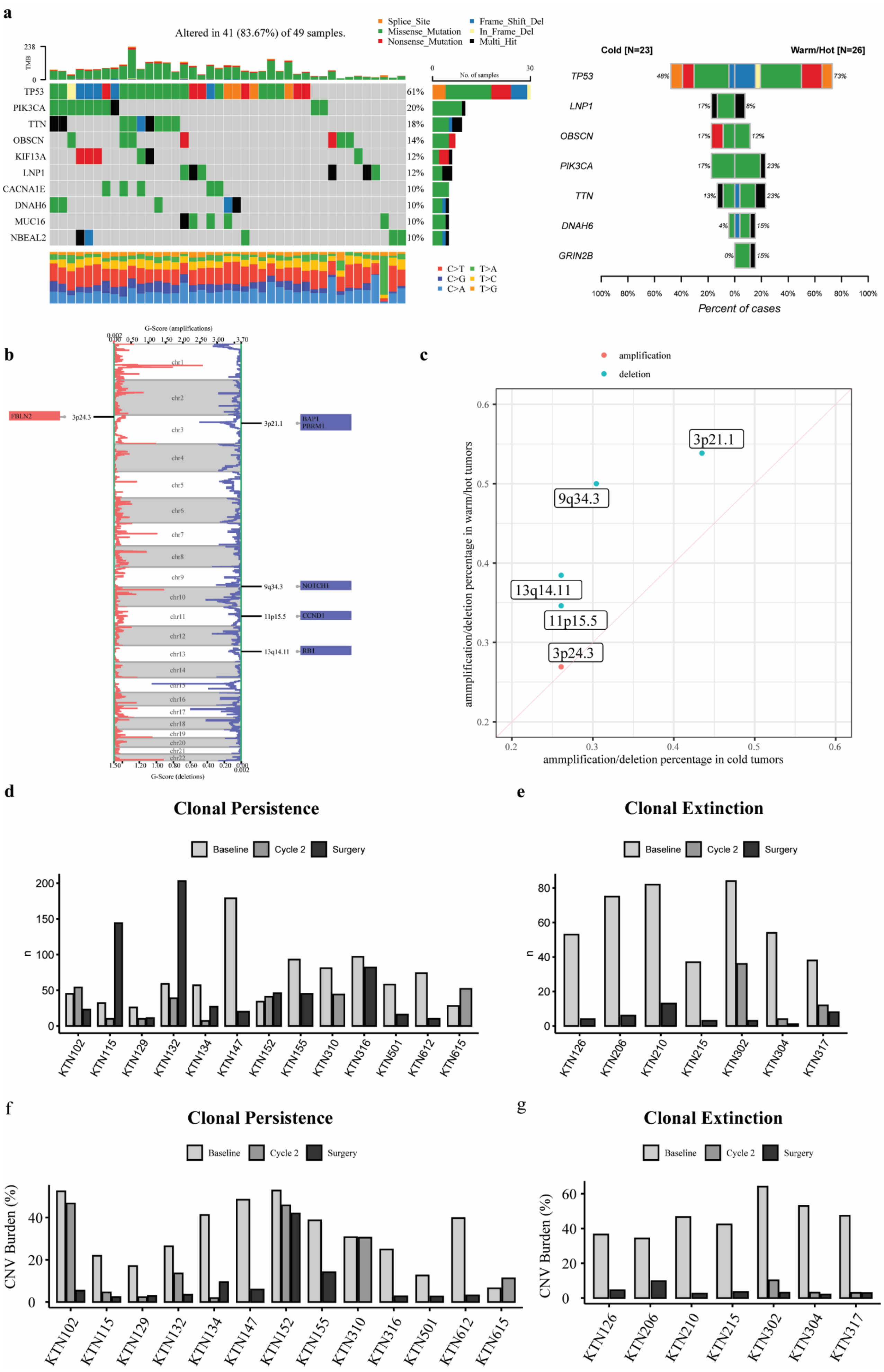

Supplementary Fig. 20 Somatic variants in longitudinal samples from PROMIX. a Summary of the somatic variants in each sample as a stacked barplot and variant types, and frequency of mutation across immune state**. b** Regions of gain and loss delineated by GISTIC analysis. Significance is reported as false discovery rate-corrected q-value. Known breast cancer suppressor genes and proto-oncogenes defined in COSMIC were annotated. **c** The percentage of somatic copy number alteration events between cold (x axis) and warm/hot tumors (y axis). **d,e** The barplot shows the number of coding non-synonymous SNV and Indel within longitudinal samples on clonal persistence and extinction group. **f,g** The barplot shows the number of copy number alterations within longitudinal samples on clonal persistence and extinction group.

Supplementary Fig. 21

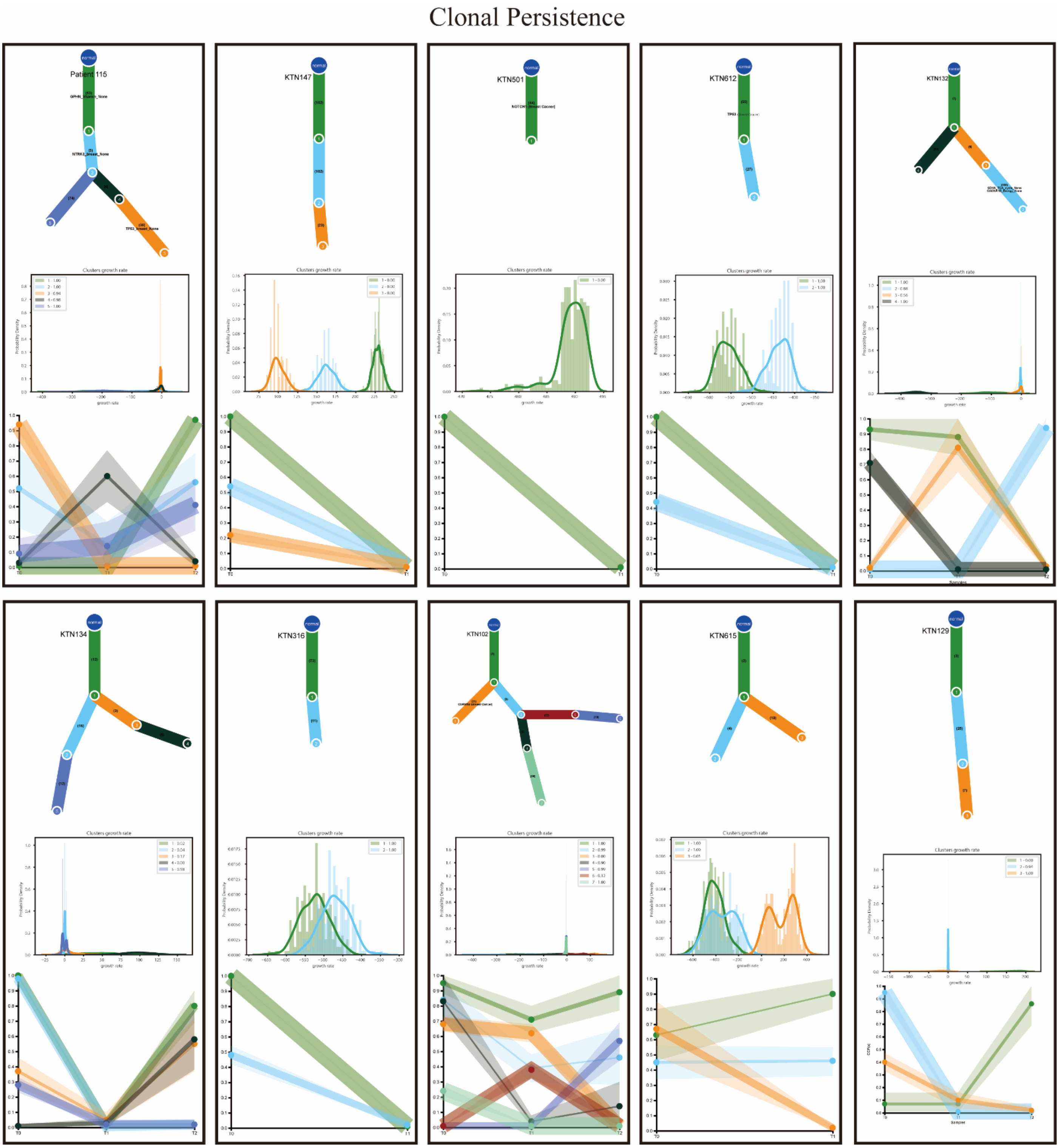

Supplementary Fig. 21 Subclones with growth advantage relative to their parent that contain known drivers in patients with clonal persistence. Results from PhylogicNDT analysis include: most likely phylogenetic tree (top); permutations of sSNVs during tree construction yielding posterior CCFs of the clusters (with 95% credible intervals) (middle); and growth rates relative to parental clones (bottom). Significance of differential growth rate (ΔGR > 0) was estimated based on the MCMC. MCMC, Markov Chain Monte Carlo.

Supplementary Fig. 22

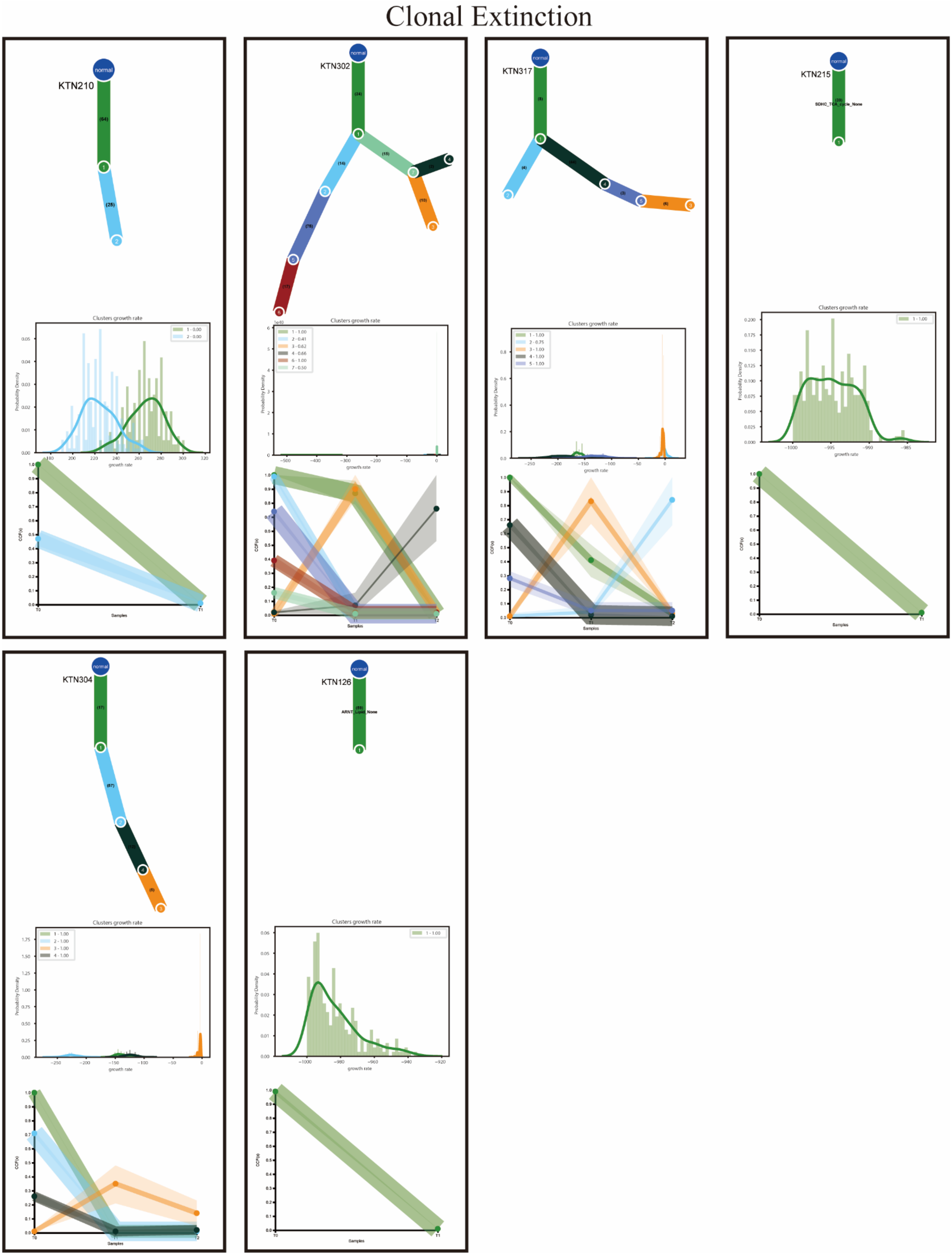

Supplementary Fig. 22 Subclones with growth advantage relative to their parent that contain known drivers in patients with clonal extinction. Results from PhylogicNDT analysis include:

most likely phylogenetic tree (top); permutations of sSNVs during tree construction yielding posterior CCFs of the clusters (with 95% credible intervals) (middle); and growth rates relative to parental clones (bottom). Significance of differential growth rate ($\Delta GR > 0$) was estimated based on the MCMC. MCMC, Markov Chain Monte Carlo.

Supplementary Table

Supplementary Table1. Clinicopathologic characteristics of BC patients from PROMIX trial.

Factor	N (%)
**Age (mean (SD))**	50.00 (9.9)
**Menopausal status**	
Premenopausal	90 (60.0)
Premenopausal (0-5 years)	17 (11.3)
Premenopausal (more than 5 years)	42 (28.0)
Missing	
**Histologic Type**	
IDC	108 (72.0)
ILC	22 (14.7)
Other	15 (10.0)
Missing	5 (3.3)
**Histologic Grade**	
I	4 (2.7)
II	46 (30.7)
III	33 (22.0)
Missing	67 (44.7)
**KI67 percentage (mean (SD))**	36.0 (24.8)
**ER status**	
Negative	41 (27.3)
Positive	107 (71.3)
Missing	2 (1.3)
**PR status**	
Negative	64 (42.7)
Positive	84 (56.0)
Missing	2 (1.3)
**Subtype**	
LumA	48 (32.0)
LumB	62 (41.3)
TNBC	38 (25.3)
Missing	2 (1.3)
**Clinical Stage**	
0	4 (2.7)
I	35 (23.3)
II	62 (41.3)
III	39 (26.0)
Missing	10 (6.7)
**Response after two cycles of NAC**	
Complete response	5 (3.3)
Partial response	64 (42.7)
Stable disease	74 (49.3)
Progressive disease	4 (2.7)
Missing	3 (2.0)
**pCR status**	
No	129 (86.0)
Yes	20 (13.3)
Missing	1 (0.7)
**Recurrence**	
No	127 (84.7)
Yes	23 (15.3)

**DFS, month**	76.80 (33.97)

Abbreviations: SD, standard deviation; IDC, invasive ductal carcinoma; ILC, invasive lobular carcinoma; IHC, immunohistochemistry; NAC**,** neoadjuvant chemotherapy; ER, estrogen receptor; PR, progesterone receptor; TNBC, triple negative breast cancer; pCR, pathologic complete response; DFS, disease-free survival.

Supplementary Table2. Clinicopathologic characteristics of all BC cohorts included in this study.

Clinicopathologic Variable	PROMIX	South Korean NAC cohort	Oslo2 cohort	scRNA-seq Cohort
**N**	149	86	39	21
**Age, mean (SD)**	50 (9.9)	NA	NA	57 (13)
**Baseline Tumor Size (%), cm**				
<2	0	9 (10.5%)	NA	3 (14.3%)
2-5	52 (34.9)	54 (62.8%)	NA	10 (47.6%)
>5	96 (64.4)	19 (22.1%)	NA	7 (33.3%)
Missing	1 (0.7%)	4 (4.7%)	NA	1 (4.8%)
**Lymph Node Metastasis**				
No/Missing	130 (87.2%)	21 (24.4%)	NA	8 (38.0%)
Yes	20 (12.8%)	65 (75.6%)	NA	13 (61.9%)
**Subtype (%)**				
HR+HER2-	111 (74.5%)	28 (32.6%)	25 (64.1%)	12(57.1%)
TNBC	38 (25.5%)	58 (67.4%)	10 (25.6%)	9 (42.9%)
Missing	0	0	4 (10.3%)	0
**pCR (%)**				
No	129 (86.6%)	61 (71.0%)	/	/
Yes	20 (13.4%)	25 (29.0%)	/	/

Abbreviations: NAC**,** neoadjuvant chemotherapy; HR, hormone receptor; HER2, human epidermal growth factor receptor 2; TNBC, triple negative breast cancer; pCR, pathologic complete response.

Supplementary Table3. Detailed data attached to Figure2.

	Cold (n=100)	Warm (n=118)	Hot (n=57)	P value
**Timepoint**				
Pre-treatment	51 (51.0)	50 (42.4)	21 (36.8)	0.50 [a]
On-treatment	27 (27.0)	36 (30.5)	19 (33.3)	
Post-treatment	22 (22.0)	32 (27.1)	17 (29.8)	
**Tumor Cellularity**				
<20%	14 (14.0)	22 (18.6)	11 (19.3)	0.73 [a]
20-50%	18 (18.0)	19 (16.1)	9 (15.8)	
>50%	32 (32.0)	36 (30.5)	12 (21.1)	
Missing	36 (36.0)	41 (34.7)	25 (43.9)	
**TIL Density**				
<10%	44 (44.0)	39 (33.1)	11 (19.3)	**0.01** [a]
10-50%	18 (18.0)	25 (21.2)	12 (21.1)	
>50%	2 (2.0)	13 (11.0)	9 (15.8)	
Missing	36 (36.0)	41 (34.7)	25 (43.9)	
**pCR**				
No	93 (93.0)	106 (89.8)	41 (71.9)	**0.0003**[a]
Yes	7 (7.0)	12 (10.2)	16 (28.1)	
**Subtype (%)**				
HR+	87 (87.0)	83 (70.3)	35 (61.4)	**0.00002** [a]
TNBC	13 (13.0)	35 (29.7)	22 (38.6)	
**PAM50**				
LumA	44 (44.0)	28 (23.7)	8 (14.0)	**0.000008** [b]
LumB	27 (27.0)	30 (25.4)	9 (15.8)	
HER2 enriched	1 (1.0)	9 (7.6)	8 (14.0)	
Basal	11 (11.0)	28 (23.7)	22 (38.6)	
Normal	17 (17.0)	23 (19.5)	10 (17.5)	
**Thorsson's Immune Subtype**				
IFN−γ dominant	59 (59.0)	85 (72.0)	46 (80.7)	**0.001** [b]
Inflammatory	31 (31.0)	29 (24.6)	10 (17.5)	
TGF−ß dominant	9 (9.0)	0 (0.0)	0 (0.0)	
Lymphocyte depleted	0 (0.0)	4 (3.4)	1 (1.8)	
Wound healing	1 (1.0)	0 (0.0)	0 (0.0)	
**Immune Cell Fraction, mean (SD)**				
CD4 T cells	0.02 (0.05)	0.02 (0.06)	0.02 (0.04)	0.865 [c]
CD8 T cells	0.14 (0.09)	0.16 (0.09)	0.20 (0.09)	**0.000002** [c]
Treg cells	0.02 (0.03)	0.03 (0.03)	0.03 (0.04)	0.23 [c]
B cells	0.07 (0.02)	0.07 (0.02)	0.08 (0.03)	**0.02** [c]
Monocytes	0.01 (0.03)	0.00 (0.01)	0.00 (0.01)	0.07 [c]
Macrophage M1	0.10 (0.07)	0.13 (0.07)	0.15 (0.07)	**8.095e-09**[c]
Macrophage M2	0.37 (0.12)	0.31 (0.11)	0.23 (0.10)	**0.0001**[c]
NK cells	0.08 (0.05)	0.07 (0.05)	0.06 (0.05)	0.08 [c]
Dendritic cells	0.10 (0.04)	0.12 (0.04)	0.13 (0.04)	**0.00002** [c]
Neutrophils	0.02 (0.05)	0.02 (0.06)	0.02 (0.04)	0.87 [c]

[a] P value (two-sided) was calculated from Chi-Square test.

[b] P value (two-sided) was calculated from Fisher's exact test.

[c] P value (two-sided) was calculated from Kruskal-Wallis test.

Supplementary Table4. Multivariate HRs or ORs (95% CIs) for DFS or pCR by immune state.

Group	Cold	P value	Warm	P value	Hot	P value
**DFS**						
**Pre-treatment**						
**All** (n)	51		50		21	
Event (n)	11		23		9	
Multivariable-adjusted HR (95% CI)[a]	1.00 (reference)	NA	1.76 [0.82, 3.76]	0.15	1.71 [0.69, 4.22]	0.25
**Luminal** (n)	47		33		14	
Event (n)	9		15		5	
Multivariable-adjusted HR (95% CI)[b]	1.00 (reference)	NA	2.18 [0.92, 5.15]	0.08	1.58 [0.52, 4.84]	0.42
**TN** (n)	4		17		7	
Event (n)	2		8		4	
Multivariable-adjusted HR (95% CI)[b]	1.00 (reference)	NA	1.33 [0.26, 6.68]	0.73	2.33 [0.40, 13.64]	0.35
**On-treatment**						
**All** (n)	27		36		19	
Event (n)	6		16		8	
Multivariable-adjusted HR (95% CI)[a]	1.00 (reference)	NA	1.87 [0.70, 4.98]	0.21	1.25 [0.36, 4.38]	0.72
**Luminal** (n)	25		24		8	
Event (n)	6		9		4	
Multivariable-adjusted HR (95% CI)[b]	1.00 (reference)	NA	1.47 [0.52, 4.18]	0.47	2.29 [0.61, 8.65]	0.22
**TN** (n)	2		12		11	
Event (n)	0		7		4	
Multivariable-adjusted HR (95% CI)[b]	1.00 (reference)	NA	Infinite	NA	Infinite	NA
**pCR**						
**Pre-treatment**						
**All** (n)	51		50		21	
pCR (n)	4		6		7	
Multivariable-adjusted OR (95% CI)[c]	1.00 (reference)	NA	1.00 [0.87, 1.15]	0.98	**1.24 [1.04, 1.48]**	**0.02**
**Luminal** (n)	47		33		14	
pCR (n)	4		1		4	
Multivariable-adjusted OR (95% CI)[d]	1.00 (reference)	NA	0.95 [0.83, 1.08]		**1.22 [1.03, 1.46]**	**0.03**
**TN** (n)	4		17		7	
pCR (n)	0		5		3	
Multivariable-adjusted OR (95% CI)[d]	1.00 (reference)	NA	1.42 [0.84, 2.42]	0.21	1.51 [0.84, 2.71]	0.18
**On-treatment**						
**All** (n)	27		36		19	

pCR (n)	1		4		7	
Multivariable-adjusted OR (95% CI)[c]	1.00 (reference)	NA	1.07 [0.89, 1.28]	0.46	**1.39 [1.11, 1.73]**	**0.005**
**Luminal** (n)	25		24		8	
pCR (n)	0		2		4	
Multivariable-adjusted OR (95% CI)[d]	1.00 (reference)	NA	1.09 [0.94, 1.26]	0.25	**1.64 [1.33, 2.02]**	**0.00002**
**TN** (n)	2		12		11	
pCR (n)	1		2		3	
Multivariable-adjusted OR (95% CI)[d]	1.00 (reference)	NA	0.53 [0.25, 1.15]	0.12	0.63 [0.29, 1.35]	0.25

[a]Stratified by breast cancer subtype (luminal and TN) due to PH assumption violation and adjusted for tumor size (<2cm, 2-5cm, >5cm), lymph node status (metastasis, no metastasis), and mRNA MKi67.

[b]Adjusted for tumor size (<2cm, 2-5cm, >5cm), lymph node status (metastasis, no metastasis), and mRNA MKi67.

[c]Adjusted for tumor size (<2cm, 2-5cm, >5cm) and lymph node status (metastasis, no metastasis).

[d]Adjusted for breast cancer subtype (luminal, TN), tumor size (<2cm, 2-5cm, >5cm) and lymph node status (metastasis, no metastasis).

P values (two-sided) were derived from multivariable-adjusted logistic regression or Cox regression models.

Supplementary Table5. Multivariate HRs or ORs (95% CIs) for DFS or pCR by immunometabolic subtypes.

Group	Multivariable-adjusted HR/OR (95% CI)	P value
DFS[a] Upregulated vs. downregulated/Hot vs. Cold		
**TME**	1.32 [0.60, 2.90]	0.49
**Amino Acid (TC)**	1.11 [0.51, 2.39]	0.79
**Amino Acid (Bulk)**	1.14 [0.51, 2.58]	0.75
**Lipid (TC)**	1.49 [0.48, 4.62]	0.49
**Lipid (Bulk)**	2.83 [0.58, 13.71]	0.20
**Carbohydrate (TC)**	**2.62 [1.07, 6.44]**	**0.04**
**Carbohydrate (Bulk)**	1.84 [0.64, 5.31]	0.26
**TCA cycle (TC)**	**2.89 [1.16, 7.21]**	**0.02**
**TCA cycle (Bulk)**	0.96 [0.36, 2.58]	0.94
**Energy (TC)**	1.54 [0.35, 6.65]	0.57
**Energy (Bulk)**	1.16 [0.22, 6.12]	0.87
**Nucleotide (TC)**	1.17 [0.42, 3.31]	0.76
**Nucleotide (Bulk)**	1.00 [0.83, 1.21]	0.99
**Vitamin/co-factor (TC)**	1.34 [0.48, 3.76]	0.57
**Vitamin/co-factor (Bulk)**	1.03 [0.82, 1.28]	0.81
pCR[b] Upregulated vs. downregulated/Hot vs. Cold		
**TME**	**1.33 [1.15, 1.54]**	**0.001**
**Amino Acid (TC)**	**0.87 [0.76, 1.01]**	**0.07**
**Amino Acid (Bulk)**	1.02 [0.92, 1.14]	0.69
**Lipid (TC)**	0.94 [0.77, 1.13]	0.50
**Lipid (Bulk)**	**0.77 [0.59, 1.00]**	**0.06**
**Carbohydrate (TC)**	0.95 [0.77, 1.17]	0.61
**Carbohydrate (Bulk)**	0.93 [0.77, 1.12]	0.46
**TCA cycle (TC)**	**0.87 [0.74, 1.03]**	**0.10**
**TCA cycle (Bulk)**	0.87 [0.71, 1.06]	0.16
**Energy (TC)**	0.90 [0.70, 1.15]	0.41
**Energy (Bulk)**	0.83 [0.65, 1.05]	0.14
**Nucleotide (TC)**	**0.77 [0.61, 0.98]**	**0.04**
**Nucleotide (Bulk)**	3.40 [0.41, 28.44]	0.26
**Vitamin/co-factor (TC)**	0.89 [0.75, 1.05]	0.18
**Vitamin/co-factor (Bulk)**	2.85 [0.50, 16.29]	0.24

[a]Stratified by breast cancer subtype (luminal and TN) due to PH assumption violation and adjusted for tumor size (<2cm, 2-5cm, >5cm) and lymph node status (metastasis, no metastasis).

[b]Adjusted for breast cancer subtype (luminal, TN), tumor size (<2cm, 2-5cm, >5cm) and lymph node status (metastasis, no metastasis).
P values (two-sided) were derived from multivariable-adjusted logistic regression models.

Supplementary Table6. Baseline clinicopathologic characteristics of 69 BC patients divided by dynamic TME change.

	**Negative TME change**	**Positive TME change**	**P value**
**N**	24	45	
**Age (mean (SD))**	51.53 (8.95)	47.18 (10.48)	0.09 [a]
**Histologic grade (%)**			
I	0 (0.0)	1 (2.2)	0.1 [b]
II	10 (41.7)	9 (20.0)	
III	3 (12.5)	16 (35.6)	
Missing	11 (45.8)	19 (42.2)	
**Subtype (%)**			
LumA	12 (50.0)	8 (17.8)	**0.004** [b]
LumB	9 (37.5)	18 (40.0)	
TNBC	2 (8.3)	19 (42.2)	
Missing	1 (4.2)	0 (0.0)	
**Clinical Stage (%)**			
0	1 (4.2)	1 (2.2)	0.07 [b]
I	3 (12.5)	18 (40.0)	
II	15 (62.5)	14 (31.1)	
III	5 (20.8)	10 (22.2)	
Missing	0 (0.0)	2 (4.4)	
**pCR (%)**			
No	23 (95.8)	35 (77.8)	0.1 [b]
Yes	1 (4.2)	10 (22.2)	
**Radiologic response after 2 cycles (%)**			
No	19	5	0.03 [c]
Yes	22	23	

[a] P value (two-sided) was calculated from Wilcoxon rank-sum test.
[b] P value (two-sided) was calculated from Fisher's exact test.
[c] P value (two-sided) was calculated from Chi-Square test.

Supplementary Table7. Multivariate ORs (95% CIs) for response after two cycles of NAC by integrated immunometabolic subtypes.

Group	Group1[a]	P value	Group2[b]	P value	Group3[c]	P value	Group3[d]	P value
**Amino Acid**								
Multivariable-adjusted OR[e] (95% CI)	1.00 (reference)	NA	**0.72 [0.54, 0.97]**	**0.04**	0.80 [0.54, 1.19]	0.27	**0.70 [0.49, 1.01]**	**0.06**
**Lipid**								
Multivariable-adjusted OR[e] (95% CI)	1.00 (reference)	NA	0.87 [0.66, 1.15]	0.34	**0.68 [0.48, 1.05]**	**0.10**	1.27 [0.86, 1.88]	0.23
**Carbohydrate**								
Multivariable-adjusted OR[e] (95% CI)	1.00 (reference)	NA	0.81 [0.61, 1.07]	0.14	0.65 [0.36, 1.18]	0.16	0.95 [0.69, 1.30]	0.74
**TCA cycle**								
Multivariable-adjusted OR[e] (95% CI)	1.00 (reference)	NA	0.84 [0.63, 1.11]	0.23	**0.67 [0.42, 1.08]**	**0.10**	1.07 [0.67, 1.71]	0.76
**Energy**								
Multivariable-adjusted OR[e] (95% CI)	1.00 (reference)	NA	0.81 [0.61, 1.07]	0.14	**0.62 [0.40, 0.95]**	**0.03**	**0.72 [0.50, 1.04]**	**0.09**
**Nucleotide**								
Multivariable-adjusted OR[e] (95% CI)	1.00 (reference)	NA	**0.79 [0.61, 1.04]**	**0.10**	0.63 [0.24, 1.68]	0.36	0.90 [0.61, 1.33]	0.60
**Vitamin/co-factor**								
Multivariable-adjusted OR[e] (95% CI)	1.00 (reference)	NA	0.85 [0.65, 1.12]	0.26	**0.67 [0.42, 1.07]**	**0.10**	1.30 [0.71, 2.36]	0.40

[a]Positive TME change plus downregulated metabolitic phenotype change
[b]Negative TME change plus downregulated metabolitic phenotype change
[c]Negative TME change plus upregulated metabolitic phenotype change
[d]Positive TME change plus upregulated metabolitic phenotype change
[e]Adjusted for breast cancer subtype (luminal, TN), tumor size (<2cm, 2-5cm, >5cm) and lymph node status (metastasis, no metastasis).
All P values (two-sided) were derived from multivariable-adjusted logistic regression models.

Supplementary Table8. List of antibodies and experimental conditions used for the multiplex fluorescent IHC methods.

Order	Antigen retrieval*	Marker	Clone	Cat No/Lot	RRID	Host Species	Dilution	Company
1	pH9	CD68	PG-M1	M0876	AB_2074844	Mouse	1:1000	Agilent
2	pH6	CD163	10D6	NCL-L-CD163	AB_2756375	Mouse	1:100	Novocastra
3	pH6	CD4	4B12	M7310	AB_2728838	Mouse	1:100	Agilent
4	pH6	CD8a	C8/144B	14-0085-82	AB_11150240	Mouse	1:200	Thermo Fisher
5	pH6	CD20	L26	M0755	AB_2282030	Mouse	1:1000	Agilent
6	pH6	FoxP3	D6O8R	12653	AB_2797979	Rabbit	1:300	Cell Signaling
7	pH6	Cytokeratin	AE1/AE3	M3515	AB_2132885	Mouse	1:400	Dako
8	-	DAPI	-			-	-	Perkin Elmer

*Antigen retrieval performed in an automated Leica Bond RXm Research Stainer at 95 ^{0}C, 20min. The ImmPRESS® HRP Anti-Mouse IgG (Peroxidase) (Cat. No: MP-7402-50), Anti-Rabbit IgG (Peroxidase) Polymer Detection Kits, made in Horse (Cat No: MP-7401-50) (Vector Laboratories) and Opal Polymer HRP (Mouse + Rabbit, ARH1001EA, Perkin Elmer) were used as secondary antibodies.

Supplementary Table9. List of antibodies used for the multiplex immunofluorescent NK cell panel.

	**Source**	Clone	RRID	**Identifier**
**Antibodies**				
FITC anti-human FcεRI γ subunit	Milli-Mark	Polyclonal	AB_11203492	Cat#FCABS400F
PE anti-human NKG2C	BioLegend	REA205	AB_2751835	Cat#375004; Clone: S19005E
APC anti-human CD57	BioLegend	HNK-1	AB_2562757	Cat#369610; Clone: HNK-1
Anti-human CD3	Abcam	SP7	AB_443425	Cat#ab16669; Clone: SP7
Dylight 405 anti-human PAN-CK	NOVUS,biotechne	PAN-CK(Cocktail)	AB_3095533	Cat#NBP2-76425V
Alexa Fluor 532 anti-human CD56	NOVUS,biotechne	123C3.D5	AB_3095532	Cat#NBP2-33132AF532
Alexa Fluor 594 goat anti-rabbit IgG (H+L)	Thermo Fisher Scientific			Cat#A11037
**Immunfluorescence Staining Reagents**				
Antigen Unmasking Solution (Citrate-based)	Vector Laboratories	/	/	Cat#H-3300
Goat Serum (Normal)	Agilent Dako	/	/	Cat#X0907
ProLong^{TM} Diamond Antifade Mountant	Thermo Fisher Scientific	/	/	Cat#P36961
Hochest 33342	Thermo Fisher Scientific	/	/	Cat#62249

Reference:

1 Zerdes, I. *et al.* Interplay between copy number alterations and immune profiles in the early breast cancer Scandinavian Breast Group 2004-1 randomized phase II trial: results from a feasibility study. *NPJ Breast Cancer* **7**, 144, doi:10.1038/s41523-021-00352-3 (2021).

2 Park, Y. H. *et al.* Chemotherapy induces dynamic immune responses in breast cancers that impact treatment outcome. *Nat Commun* **11**, 6175, doi:10.1038/s41467-020-19933-0 (2020).

3 Johansson, H. J. *et al.* Breast cancer quantitative proteome and proteogenomic landscape. *Nat Commun* **10**, 1600, doi:10.1038/s41467-019-09018-y (2019).

4 Wu, S. Z. *et al.* A single-cell and spatially resolved atlas of human breast cancers. *Nat Genet* **53**, 1334-1347, doi:10.1038/s41588-021-00911-1 (2021).

5 Talevich, E., Shain, A. H., Botton, T. & Bastian, B. C. CNVkit: Genome-Wide Copy Number Detection and Visualization from Targeted DNA Sequencing. *PLoS Comput Biol* **12**, e1004873, doi:10.1371/journal.pcbi.1004873 (2016).
