# Supplementary material for: Longitudinal molecular profiling elucidates immunometabolism dynamics in breast cancer
Source: Nat Commun. 2024 May 7;15:3837. doi: 10.1038/s41467-024-47932-y (PMC11076527; doi:10.1038/s41467-024-47932-y)
Supplement: Supplementary file 3 — Description of Additional Supplementary Files [file 41467_2024_47932_MOESM3_ESM.pdf]

Description of Additional Supplementary Files

File Name: Supplementary Data 1

Description: Immune cells density derived from whole-section multiplex fluorescent immunohistochemistry (mfIHC). The number of positive cells normalized to tissue area.

File Name: Supplementary Data 2

Description: mRNA expression for tumor cells only. The data were generated by ISOpureR, including 270 samples with tumor cells.

File Name: Supplementary Data 4

Description: Differentially expressed genes across tumor-cell-based metabolic state. Given the seven tumor-cellbased metabolic expression subtypes, expression differentiation of protein-coding genes across three group were evaluated using ANOVA. Here "trend" is equal to 0, it means there is no expected three pair-wise comparison (i.e., lower expression in upregulated group, higher expression in downregulated group)

File Name: Supplementary Data 5

Description: Correlation between protein interactions of known complex members from Biogrid or CORUM in mRNA and protein.

File Name: Supplementary Data 6

Description: Pair-wise differentially expressed genes across sampling time.

File Name: Supplementary Data 7

Description: Metabolic pathways enriched within breast epithelial cells, related to figure S15A.

File Name: Supplementary Data 8

Description: Metabolic pathways enriched within immune cells, related to figure S15B.

File Name: Supplementary Data 9

Description: Metabolic pathways enriched within stromal cells and monocytes, related to figure S15C.

File Name: Supplementary Data 10

Description: Somatic single nucleotide variants (SNVs) and insertion/deletion variants (Indels) in 20 triple negative breast cancer patients from PROMIX trial.

File Name: Supplementary Data 11

Description: TCGA PanCancerAtlas immune gene set.

File Name: Supplementary Data 12

Description: Gene set mapped in the seven metabolic pathways.
