# Supplementary material for: Longitudinal molecular profiling elucidates immunometabolism dynamics in breast cancer
Source: Nat Commun. 2024 May 7;15:3837. doi: 10.1038/s41467-024-47932-y (PMC11076527; doi:10.1038/s41467-024-47932-y)
Supplement: Supplementary file 16 — Reporting Summary [file 41467_2024_47932_MOESM16_ESM.pdf]

Corresponding author(s): Theodoros Foukakis

Last updated by author(s): Mar 16, 2024

Reporting Summary

Nature Portfolio wishes to improve the reproducibility of the work that we publish. This form provides structure for consistency and transparency in reporting. For further information on Nature Portfolio policies, see our Editorial Policies and the Editorial Policy Checklist.

Statistics

For all statistical analyses, confirm that the following items are present in the figure legend, table legend, main text, or Methods section.

n/a	Confirmed	
☐	☒	The exact sample size (*n*) for each experimental group/condition, given as a discrete number and unit of measurement
☐	☒	A statement on whether measurements were taken from distinct samples or whether the same sample was measured repeatedly
☐	☒	The statistical test(s) used AND whether they are one- or two-sided<br>*Only common tests should be described solely by name; describe more complex techniques in the Methods section.*
☐	☒	A description of all covariates tested
☐	☒	A description of any assumptions or corrections, such as tests of normality and adjustment for multiple comparisons
☐	☒	A full description of the statistical parameters including central tendency (e.g. means) or other basic estimates (e.g. regression coefficient) AND variation (e.g. standard deviation) or associated estimates of uncertainty (e.g. confidence intervals)
☐	☒	For null hypothesis testing, the test statistic (e.g. *F*, *t*, *r*) with confidence intervals, effect sizes, degrees of freedom and *P* value noted<br>*Give P values as exact values whenever suitable.*
☐	☒	For Bayesian analysis, information on the choice of priors and Markov chain Monte Carlo settings
☒	☐	For hierarchical and complex designs, identification of the appropriate level for tests and full reporting of outcomes
☐	☒	Estimates of effect sizes (e.g. Cohen's *d*, Pearson's *r*), indicating how they were calculated

Our web collection on statistics for biologists contains articles on many of the points above.

Software and code

Policy information about availability of computer code

Data collection	No software or code was used to collect data
Data analysis	We used existing computational softwares as follows:<br>BCFTools (ver. 1.9; https://samtools.github.io/bcftools/bcftools.html)<br>bwa (ver. 0.7.17, https://github.com/lh3/bwa)<br>bwa-mem (ver. 2 2.0, https://github.com/lh3/bwa)<br>CopyKAT (ver. 1.0.6; https://github.com/navinlabcode/copykat)<br>CNVkit (ver. 0.9.6; https://cnvkit.readthedocs.io/en/stable/)<br>ESTIMATE (ver. 1.0.13; https://r-forge.r-project.org/R/?group_id=2237)<br>Fiji ImageJ (ver. 2.9.0; https://imagej.net/software/fiji/)<br>FastQC (ver. 0.11.9; https://www.bioinformatics.babraham.ac.uk/projects/fastqc/)<br>fgbio (ver. 1.1.0; http://fulcrumgenomics.github.io/fgbio/)<br>GISTIC2 (ver. 2; https://github.com/broadinstitute/gistic2)<br>GATK4-spark (ver. 4.1.7.0; https://gatk.broadinstitute.org/hc/en-us/articles/360035890591-Spark)<br>ggplot2 (ver. 3.3.0; https://cran.r-project.org/web/packages/ggplot2/index.html)<br>Immunedeconv (ver. 2.1.0; https://github.com/omnideconv/immunedeconv)<br>ISOpureR (ver. 1.1.3; https://cran.r-project.org/web/packages/ISOpureR/index.html)<br>MultiQC (ver. 1.8; https://multiqc.info/)<br>nf-core/sarek (ver. 2.7.0; https://nf-co.re/sarek/2.7)<br>PhylogicNDT (ver. 1.0; https://github.com/broadinstitute/PhylogicNDT)<br>QuanTIseq (ver. 1.6.0; https://icbi.i-med.ac.at/software/quantiseq/doc/)

Qualimap (ver. 2.2.2d; http://qualimap.conesalab.org/)
R (ver. 4.0.1; https://www.r-project.org/)
Seurat (ver. 4.0.1; https://satijalab.org/seurat/)
SAMBLASTER (ver. 0.1.24; https://github.com/GregoryFaust/samblaster)
Samtools (ver. 1.9; http://www.htslib.org/)
snpEff (ver. 4.3.1t; https://pcingola.github.io/SnpEff/)
Strelka2 (ver. 2.9.10; https://github.com/Illumina/strelka)
PureCN (ver. 1.2.0; https://bioconductor.org/packages/PureCN/)
pigz (ver. 2.3.4; https://zlib.net/pigz/)
Trim Galore (ver. 0.6.5; https://www.bioinformatics.babraham.ac.uk/projects/trim_galore/)
VCFanno (ver. 0.3.2; https://github.com/brentp/vcfanno)
VCFtools (ver. 0.1.16; https://vcftools.sourceforge.net/)
VEP (ver. 99.2; https://www.ensembl.org/info/docs/tools/vep/index.html)
Monocle3 (ver.1.3.4; https://cole-trapnell-lab.github.io/monocle3/)

The code used to determine tumor cell-based metabolic phenotype (i.e. downregulated, neutral, upregulated) is available at https://github.com/WangKang-Leo/PureMeta.

For manuscripts utilizing custom algorithms or software that are central to the research but not yet described in published literature, software must be made available to editors and reviewers. We strongly encourage code deposition in a community repository (e.g. GitHub). See the Nature Portfolio guidelines for submitting code & software for further information.

Data

Policy information about availability of data

All manuscripts must include a data availability statement. This statement should provide the following information, where applicable:

- Accession codes, unique identifiers, or web links for publicly available datasets
- A description of any restrictions on data availability
- For clinical datasets or third party data, please ensure that the statement adheres to our policy

The mass spectrometry proteomics raw data generated in this study have been deposited in the ProteomeXchange Consortium with the data set identifier PXD039529 (URL: https://repository.jpostdb.org/entry/JPST001987). The microarray-based gene expression data are deposited in GEO under accession code GSE87455. The WES and snRNA-seq raw data have been deposited in NCBI Sequence Read Archive under accession SRA: SRP114962 (URL: https://www.ebi.ac.uk/ena/browser/view/PRJNA396019). The databases providing gene (signatures), protein or cell line reference: TCGA PanCancerAtlas (https://cri-iatlas.org/), CORUM-3.0 (http://mips.helmholtz-muenchen.de/corum), BIOGRID-4.4.201 (https://downloads.thebiogrid.org/BioGRID), Cancer Gene Census (CGC) (https://cancer.sanger.ac.uk/census), ProteinAtlas (http://www.proteinatlas.org/humanproteome/druggable), and DepMap 21Q2 (https://depmap.org/portal/). Source data (Figshare DOI: https://doi.org/10.6084/m9.figshare.22687246) are provided with this paper.

Research involving human participants, their data, or biological material

Policy information about studies with human participants or human data. See also policy information about sex, gender (identity/presentation), and sexual orientation and race, ethnicity and racism.

Reporting on sex and gender	No gender specific analyses were performed, because all breast cancer patients included were female. The trial recruited female patients, because male breast cancers are not common (about 1%).
Reporting on race, ethnicity, or other socially relevant groupings	No race, ethnicity, or other socially relevant groupings were provided, because the study recruited Swedish patients locally.
Population characteristics	Our cohort consists of 149 patients, whose characteristics were shown in Supplementary Table1.
Recruitment	PROMIX trial (ClinicalTrials.gov identifier NCT00957125) enrolled patients with locally advanced (tumor size>20 mm) HER2-negative breast cancer.
Ethics oversight	The clinical study and correlative analyses were approved by the Ethics Committee at Karolinska University Hospital, 2007/1529–31/2 and patients provided written informed consent for their participation in the clinical trial and for translational research.

Note that full information on the approval of the study protocol must also be provided in the manuscript.

Field-specific reporting

Please select the one below that is the best fit for your research. If you are not sure, read the appropriate sections before making your selection.

☒ Life sciences ☐ Behavioural & social sciences ☐ Ecological, evolutionary & environmental sciences

For a reference copy of the document with all sections, see nature.com/documents/nr-reporting-summary-flat.pdf

Life sciences study design

All studies must disclose on these points even when the disclosure is negative.

Sample size	Assuming that 120 patients will be operated after the sixth treatment cycle (excluding early cases of PD), and that 25% will be free from tumour (pCR), the power is 80% for detection of a true difference (e.g. for a molecular marker) of 0.6 SD between the number of patients with tumours in pCR compared to those without. In order to detect a true difference of 1 SD, the power is over 99%. Hence, this trial planned to enroll a total of 151 patients.
Data exclusions	No data were excluded from reporting.
Replication	Reproducibility is ensured through full availability of both code and required data.
Randomization	Non-randomized single arm clinical trial.
Blinding	The investigators were blinded to the clinical data during experiments, pathologic assessment, data collection and analysis.

Reporting for specific materials, systems and methods

We require information from authors about some types of materials, experimental systems and methods used in many studies. Here, indicate whether each material, system or method listed is relevant to your study. If you are not sure if a list item applies to your research, read the appropriate section before selecting a response.

Materials & experimental systems

n/a	Involved in the study	
☐	☒	Antibodies
☐	☒	Eukaryotic cell lines
☒	☐	Palaeontology and archaeology
☒	☐	Animals and other organisms
☐	☒	Clinical data
☒	☐	Dual use research of concern
☒	☐	Plants

Methods

n/a	Involved in the study	
☒	☐	ChIP-seq
☒	☐	Flow cytometry
☒	☐	MRI-based neuroimaging

Antibodies

Antibodies used

Antibodies used for multiplex immunofluorescence are listed in Methods and in Supplementary Tables 8 and 9

Validation

Anti-Human CD68, Clone PG-M1, is intended for use in immunohistochemistry (IHC). As It is stated in the manufacturer's webpage (https://shorturl.at/ciw37), the PG-M1 antibody (unlike other CD68 antibodies) detects a fixative-resistant epitope on the macrophage-restricted form of the CD68 antigen (Falini B, et al. PG-M1: A new monoclonal antibody directed against a fixative-resistant epitope on the macrophage- restricted form of the CD68 molecule. Am J Pathol 1993;142:1359-72)

According to the manufacturer the monoclonal Mouse Anti-Human CD163, Clone 10D6 is intended for the qualitative identification by light microscopy of human CD163 antigen in FFPE sections -as stated in the respective datashett (https://shorturl.at/gmvGY). In normal tissues clone 10D6 detected the CD163 protein in the membrane and cytoplasm of monocytes and macrophages (Lau SK, et al. CD163: a specific marker of macrophages in paraffin-embedded tissue samples. American Journal of Clinical Pathology. 2004; 122(5):794-801)

According to the manufacturer the monoclonal Mouse Anti-Human CD4, Clone 4B12, is intended for use in IHC in FFPE tissue sectionns and labels thymocytes and T-helper cells. According to manufacturer's homepage (https://shorturl.at/htxEK), the positive control tissue include tonsil and liver and the reaction patterns are the following: i) in tonsil, the crowded and isolated T-helper cells show a moderate to strong staining reaction while ii) in liver, the Kupffer and endothelial cells of the sinusoids show a weak to moderate staining reaction.
According to the manufacturer the monoclonal Mouse Anti-Human CD8a, Clone C8/144B is intended for use in IHC on FFPE tissue sections. It recognizes the human CD8a molecule in thymocytes, mature T cells and NK cells. According to the description in manufacturer's homepage (https://www.thermofisher.com/antibody/product/CD8a-Antibody-clone-C8-144B-Monoclonal/14-0085-82), this antibody clone has been previously has been tested by IHC and validated on FFPE human tonsil tissue
According to the manufacturer the monoclonal Mouse Anti-Human CD20, Clone L26 is intended for use in IHC. The antibody labels cells of the B-cell lineage and is a useful aid for the classification of neoplasms of B-cell derivation. In normal lymphoid tissue, the antibody labeled germinal centre cells, mantle zone lymphocytes, and scattered interfollicular lymphocytes, but not T cells, histiocytes and plasma cells (https://shorturl.at/fzKO8) (Mason DY, et al. Antibody L26 recognizes an intracellular epitope on the B-cell-associated CD20 antigen. Am J Pathol 1990;136:1215-22)
According to the manufacturer the monoclonal Rabbit Anti-Human FoxP3, Clone D6O8R recognizes endogenous levels of total FoxP3 protein, which is crucial for the development of T cells with regulatory properties (Treg). This antibody recognizes mouse FoxP3 protein and is also reactive with human FoxP3 (https://www.cellsignal.com/products/primary-antibodies/foxp3-d6o8r-rabbit-mab/12653). It has been also previously tested in non-small cell lung cancer tissue (Backman M et al. Spatial immunophenotyping of

the tumour microenvironment in non-small cell lung cancer. Eur J Cancer. 2023 May:185:40-52
According to the manufacturer the monoclonal Mouse Anti-Human Cytokeratin, Clone AE1/AE3, is intended for use in IHC and identifies two epitopes present on a majority of epithelial cytokeratins in FFPE tissue. Results aid in the classification of normal and neoplastic tissue as epithelial in origin. According to the description on the manufacturer's webpage (https://shorturl.at/lLQZ7), the antibody clones AE1/AE3 have previously tested in several poorly differentiated epithelial neoplasms, lymphomas, melanomas and sarcoma (Listrom MB, Dalton LW. Comparison of keratin monoclonal antibodies MAK-6, AE1:AE3 and CAM-5.2. Amer J Clin Pathol 1987;88(3):297-301)
According to the manufacturer, the monoclonal Rabbit Anti-Human CD3, Clone SP7 is suitable for staining normal and neoplastic T cells in formalin-fixed, paraffin-embedded tissues. According to the description on the manufacturer's webpage (https://www.abcam.com/en-se/products/primary-antibodies/cd3-epsilon-antibody-sp7-ab16669#tab=datasheet) it has been validated in mIHC, IHC-P, Flow Cyt (Intra), WB and tested in Human, Mouse, Rat samples. Cited in 415 publications.(Moussion C et al., PLoS One. 2008 Oct 6;3(10):e3331. Fig 2) doi:10.1371/journal.pone.0003331; October 6, 2008, PLoS ONE 3(10): e3331
According to the manufacturer the monoclonal Mouse Anti-Human CD56, Clone123C3.D5,can be used for Flow Cytometry,Immunocytochemistry/ Immunofluorescence, Immunohistochemistry, Immunohistochemistry-Paraffin. According to the description on the manufacturer's webpage (https://www.novusbio.com/products/ncam-1-cd56-antibody-123c3d5_nbp2-33132af532), it has been tested in Western blot, Flow, ICC/IF, IHC
According to the manufacturer the monoclonal Mouse Anti-Human CD57, Clone HNK-1 can be used for immunofluorescent staining. According to the description on the manufacturer's webpage (https://www.biolegend.com/en-gb/products/apc-anti-human-cd57-antibody-9023?GroupID=BLG8483), it has been tested by lots of studies for immunofluorescence staining. Yang C, et al. 2019. Nat Commun. 10:3931.
According to the manufacturer the monoclonal Mouse Anti-Human Pan-Cytokeratin, Clone PAN-CK (Cocktail) can be used in WB, Flow, ICC/IF, IHC, CyTOF-ready, ICC/IF (https://www.novusbio.com/products/cytokeratin-pan-antibody-pan-ck-cocktail-_nbp2-76425v#datasheet). It has been tested in ovarian cancers (Bruand M,et al. Cell-autonomous inflammation of BRCA1-deficient ovarian cancers drives both tumor-intrinsic immunoreactivity and immune resistance via STING. Cell Rep. 2021 Jul 20;36(3):109412. doi: 10.1016/j.celrep.2021.109412. PMID: 34289354
According to the manufacturer the monoclonal Mouse Anti-human PE conjugated NKG2C is suitable immunofluorescence even in fixed states (https://www.miltenyibiotec.com/US-en/products/cd159c-nkg2c-antibody-anti-human-reafinity-rea205.html#conjugate=vio-bright-r720:size=100-tests-in-200-ul)
According to the manufacturer the polyclonal Rabbit Anti-human FITC FcεRγ is suitable immunofluorescence (Milli-Mark® Anti-FcεRI Antibody, γ subunit-FITC Milli-Mark®, from rabbit | Sigma-Aldrich (sigmaaldrich.com)

Eukaryotic cell lines

Policy information about cell lines and Sex and Gender in Research

Cell line source(s)	human breast cancer cell lines:<br>BT-549 cells from ATCC (https://www.atcc.org/products/htb-122)<br>MDA-MB-231 cells from ATCC (https://www.atcc.org/products/htb-26)<br>MCF7 cells from ATCC (https://www.atcc.org/products/htb-22)<br>T-47D cells from ATCC (https://www.atcc.org/products/htb-133)
Authentication	The cell lines were not authenticated (but they were used immediately upon delivery from ATCC minimizing thus the possibility of cross-contamination).
Mycoplasma contamination	All cell lines were tested negative for mycoplasma
Commonly misidentified lines (See ICLAC register)	No commonly misidentified cell lines were used in this study.

Clinical data

Policy information about clinical studies
All manuscripts should comply with the ICMJE guidelines for publication of clinical research and a completed CONSORT checklist must be included with all submissions.

Clinical trial registration	ClinicalTrials.gov identifier NCT00957125
Study protocol	https://clinicaltrials.gov/ct2/show/NCT00957125
Data collection	Patients in this study were enrolled from September 2008 to November 2011. Recruitment Site: Sahlgrenska University Hospital Göteborg, Sweden; Lund University Hospital Lund, Sweden; Malmö General University Hospital Malmö, Sweden; Karolinska University Hospital, Dept of Oncology Stockholm, Sweden, SE-17176; County Hospital Sundsvall, Sweden; Uppsala University Hospital Uppsala, Sweden
Outcomes	Primary Outcome Measures :<br>1. Evaluation of the sensitivity and of defined diagnostic and biological procedures to detect response/non-response to neoadjuvant treatment at an early point among patients with breast cancer. [Time Frame: 6 weeks]<br>Secondary Outcome Measures :<br>1.Identification of tumour characteristics and treatment-related changes of tumour characteristics predictive of long-term prognosis. [Time Frame: 5 years]<br>2.Comparison between the standard evaluation procedures mammography, conventional ultrasound and clinical examination and functional imaging techniques and biological procedures with emphasis on detection of response at an early point of treatment.

[Time Frame: 6 weeks]
3.Studies on the addition of bevacizumab with regard to further improvement of response in tumours with stable (SD) or partial response (PR) and the impact of treatment on angiogenesis and local features of the tumour environment. [Time Frame: 6 weeks]
4.Acute toxicity [Time Frame: 6 weeks after last chemotherapy]
5.Late toxicity [Time Frame: 5 years after last chemotherapy]
